# Integration of multi-omics data and deep phenotyping provides insights into responses to single and combined abiotic stress in potato

**DOI:** 10.1093/plphys/kiaf126

**Published:** 2025-04-02

**Authors:** Maja Zagorščak, Lamis Abdelhakim, Natalia Yaneth Rodriguez-Granados, Jitka Široká, Arindam Ghatak, Carissa Bleker, Andrej Blejec, Jan Zrimec, Ondřej Novák, Aleš Pěnčík, Špela Baebler, Lucia Perez Borroto, Christian Schuy, Anže Županič, Leila Afjehi-Sadat, Bernhard Wurzinger, Wolfram Weckwerth, Maruša Pompe Novak, Marc R Knight, Miroslav Strnad, Christian Bachem, Palak Chaturvedi, Sophia Sonnewald, Rashmi Sasidharan, Klára Panzarová, Kristina Gruden, Markus Teige

**Affiliations:** Department of Biotechnology and Systems Biology, National Institute of Biology, Večna pot 121, 1000 Ljubljana, Slovenia; PSI (Photon Systems Instruments), spol. s r.o., Prumyslova 470, CZ-664 24 Drásov, Czech Republic; Plant Stress Resilience, Institute of Environmental Biology, Utrecht University, Heidelberglaan 8, 3584 CS Utrecht, The Netherlands; Laboratory of Growth Regulators, Palacký University in Olomouc & Institute of Experimental Botany AS CR, Šlechtitelů 27, Olomouc 779 00, Czech Republic; Department of Functional and Evolutionary Ecology, Molecular Systems Biology (MOSYS), University Vienna, Djerassiplatz 1, 1030 Vienna, Austria; Department of Biotechnology and Systems Biology, National Institute of Biology, Večna pot 121, 1000 Ljubljana, Slovenia; Department of Biotechnology and Systems Biology, National Institute of Biology, Večna pot 121, 1000 Ljubljana, Slovenia; Department of Biotechnology and Systems Biology, National Institute of Biology, Večna pot 121, 1000 Ljubljana, Slovenia; Laboratory of Growth Regulators, Palacký University in Olomouc & Institute of Experimental Botany AS CR, Šlechtitelů 27, Olomouc 779 00, Czech Republic; Laboratory of Growth Regulators, Palacký University in Olomouc & Institute of Experimental Botany AS CR, Šlechtitelů 27, Olomouc 779 00, Czech Republic; Department of Biotechnology and Systems Biology, National Institute of Biology, Večna pot 121, 1000 Ljubljana, Slovenia; Wageningen University and Research, Department of Plant Breeding, Droevendaalsesteeg 1, 6708 PB Wageningen, The Netherlands; Department Biologie, Lehrstuhl für Biochemie, Friedrich-Alexander-Universität Erlangen-Nürnberg, Staudstr. 5, 91058 Erlangen, Germany; Department of Biotechnology and Systems Biology, National Institute of Biology, Večna pot 121, 1000 Ljubljana, Slovenia; Mass Spectrometry Unit, Research Support Facilities, Faculty of Life Sciences, University Vienna, Djerassiplatz 1, 1030 Vienna, Austria; Department of Functional and Evolutionary Ecology, Molecular Systems Biology (MOSYS), University Vienna, Djerassiplatz 1, 1030 Vienna, Austria; Department of Functional and Evolutionary Ecology, Molecular Systems Biology (MOSYS), University Vienna, Djerassiplatz 1, 1030 Vienna, Austria; Vienna Metabolomics Center (VIME), University Vienna, Djerassiplatz 1, 1030 Vienna, Austria; Department of Biotechnology and Systems Biology, National Institute of Biology, Večna pot 121, 1000 Ljubljana, Slovenia; School for Viticulture and Enology, University of Nova Gorica, Gladni trg 8, 5271 Vipava, Slovenia; Department of Biosciences, Durham University, South Road, Durham DH1 3LE, UK; Laboratory of Growth Regulators, Palacký University in Olomouc & Institute of Experimental Botany AS CR, Šlechtitelů 27, Olomouc 779 00, Czech Republic; Wageningen University and Research, Department of Plant Breeding, Droevendaalsesteeg 1, 6708 PB Wageningen, The Netherlands; Department of Functional and Evolutionary Ecology, Molecular Systems Biology (MOSYS), University Vienna, Djerassiplatz 1, 1030 Vienna, Austria; Department Biologie, Lehrstuhl für Biochemie, Friedrich-Alexander-Universität Erlangen-Nürnberg, Staudstr. 5, 91058 Erlangen, Germany; Plant Stress Resilience, Institute of Environmental Biology, Utrecht University, Heidelberglaan 8, 3584 CS Utrecht, The Netherlands; PSI (Photon Systems Instruments), spol. s r.o., Prumyslova 470, CZ-664 24 Drásov, Czech Republic; Department of Biotechnology and Systems Biology, National Institute of Biology, Večna pot 121, 1000 Ljubljana, Slovenia; Department of Functional and Evolutionary Ecology, Molecular Systems Biology (MOSYS), University Vienna, Djerassiplatz 1, 1030 Vienna, Austria

## Abstract

Potato (*Solanum tuberosum*) is highly water and space efficient but susceptible to abiotic stresses such as heat, drought, and flooding, which are severely exacerbated by climate change. Our understanding of crop acclimation to abiotic stress, however, remains limited. Here, we present a comprehensive molecular and physiological high-throughput profiling of potato (*Solanum tuberosum*, cv. Désirée) under heat, drought, and waterlogging applied as single stresses or in combinations designed to mimic realistic future scenarios. Stress responses were monitored via daily phenotyping and multi-omics analyses of leaf samples comprising proteomics, targeted transcriptomics, metabolomics, and hormonomics at several timepoints during and after stress treatments. Additionally, critical metabolites of tuber samples were analyzed at the end of the stress period. We performed integrative multi-omics data analysis using a bioinformatic pipeline that we established based on machine learning and knowledge networks. Waterlogging produced the most immediate and dramatic effects on potato plants, interestingly activating ABA responses similar to drought stress. In addition, we observed distinct stress signatures at multiple molecular levels in response to heat or drought and to a combination of both. In response to all treatments, we found a downregulation of photosynthesis at different molecular levels, an accumulation of minor amino acids, and diverse stress-induced hormones. Our integrative multi-omics analysis provides global insights into plant stress responses, facilitating improved breeding strategies toward climate-adapted potato varieties.

## Teaser

Integrated multi-omics analysis of high-throughput phenotyping in potato reveals distinct molecular signatures of acclimation to single and combined abiotic stresses related to climate change.

## Introduction

Improving crop resilience to climate change is a major challenge of modern agriculture (Bailey-Serres et al. 2019; Rivero et al. 2022). High-yielding crop varieties including potato (*Solanum tuberosum*) are vulnerable to heat, drought, and flooding (Benitez-Alfonso et al. 2023; Zandalinas et al. 2023; Renziehausen et al. 2024; Sato et al. 2024). These environmental stresses affect plant growth, source–sink relationships, sugar and hormone metabolism, among other processes, which in turn, negatively impact product yield and nutritional status (Lal et al. 2022). Potato is particularly sensitive to waterlogging (Jovović et al. 2021), and flooding of the fields can ruin the entire harvest within a few days. Since global warming is increasing, the occurrence of such extreme weather events, crop productivity worldwide is under considerable threat (FAO 2023). To ensure future food security, there is an urgent need for sustainable farming practices including the development of stress tolerant varieties with consistent yields (Dahal et al. 2019; Lal et al. 2022).

There is already a good understanding of how plants react to single abiotic stresses, which have profound effects on plant metabolism and development. The primary effects of abiotic stress are generation of reactive oxygen species (ROS), destabilization of proteins and changes in enzyme efficiencies and membrane fluidity and integrity (Zhang et al. 2022a, 2022b). Together, these impacts reduce plant productivity through changes in photosynthetic capacity, hormone balance, transport of assimilates from source to sinks as well as transport of soil nutrients and water by the roots. In addition, species-specific vulnerabilities impact agronomic productivity, such as for instance, tuber initiation and tuber growth dynamics in potato. Potato tuber formation and growth is dependent on mobile tuberization signals produced in source leaves, such as the potato homolog of FLOWERING LOCUS T, SELF-PRUNING 6A (SP6A) (Navarro et al. 2011), which also regulates directional transport of sucrose to the developing tuber (Abelenda et al. 2019). Heat, drought, and flooding trigger strong changes in gene expression and thereby strongly interfere with the regulation of flowering and tuberization by the photoperiodic pathway. This leads to a delay in tuberization and anomalies in subsequent tuber development such as second growth and/or internal defects, which together severely impacts marketable yields of the tuber crop (Dahal et al. 2019; Lal et al. 2022).

On the other hand, the response of plants to combined stresses is unique and cannot be extrapolated from the response to the corresponding individual stresses (Mittler 2006). Considering the increasing occurrences of simultaneous or sequential abiotic stresses in the field, the relative lack of knowledge on multi-stress resilience is a major shortfall that hinders the ability to develop effective strategies for crop improvement. Accordingly, the question of how combinations of different stresses impacts plants have recently gained a lot of interest (Zandalinas et al. 2021). Several studies on combinatorial stress effects have been performed, especially studying the physiological and molecular responses to combined heat- and drought stress in potato (Demirel et al. 2020), wheat (Manjunath et al. 2023), and tomato (Zeng et al. 2024). In nature, heat and drought often occur together, resulting in different physiological responses as compared with individual stresses. For example, under heat, stomatal conductance and transpiration are increased to reduce leaf temperature, whilst under drought, stomata are closed to avoid water loss, which leads to a strongly reduced CO_2_-assimilation (Zhang and Sonnewald 2017). The final phenotypic output in a combined stress scenario greatly depends on synergistic and antagonistic interactions between stress-specific signaling and response pathways. These interactions can be regulated at various levels (gene expression to metabolism), and on different scales (cell to system), thus resulting in complex regulatory network perturbations. Therefore, as information gained by extrapolating from studies on individual stressors is limited, it is crucial to increase our understanding of crop responses in multi-stress situations.

To this end, high-throughput phenotyping (HTP) platforms and integrative omics technologies can measure molecular mechanisms at multiple levels and in multiple processes simultaneously. This can help us obtain a comprehensive understanding of the intricate dynamics of plant-environment interactions (Yang et al. 2020; Hall et al. 2022; Zhang et al. 2022a, 2022b). Here, advanced data integration pipelines can aid with unbiased integration and systematic extraction of biological knowledge from large multi-omics datasets (Cembrowska-Lech et al. 2023). However, despite the increasing application of high-throughput approaches in agricultural and plant research, only a handful of studies have addressed the problem of data integration from comprehensive multi-omics data (Jamil et al. 2020). Therefore, to enable molecular insights across various system levels and disentangle the intricate physiological and molecular crosstalk in the context of non-additive effects of different stress combinations, it is imperative to develop and apply multi-omics integrative approaches that leverage statistics, machine learning, and graph theory.

In this study, we aimed to increase knowledge on multiple abiotic stress responses of potato plants and to integrate this into a complex knowledge network. Therefore, a comprehensive assessment of potato responses to single and combined heat, drought, and waterlogging stress was performed. Using the cv. Désirée, a widely used moderately stress-resistant potato cultivar, we monitored dynamic changes in morphological, physiological, as well as biochemical and molecular responses under stress conditions. With the application of HTP, multi-omics technologies, prior knowledge, and multi-level integration approaches, we identified important molecular signatures, unique to single and different stress combinations. These results can guide the development of diagnostic markers for rapid detection of stress, allowing for earlier agricultural interventions to enhance plant resilience toward abiotic stress and development of marker-assisted breeding programs for climate-resilient crops (Weckwerth et al. 2020; Mishra et al. 2024).

## Results

**Figure 1.**
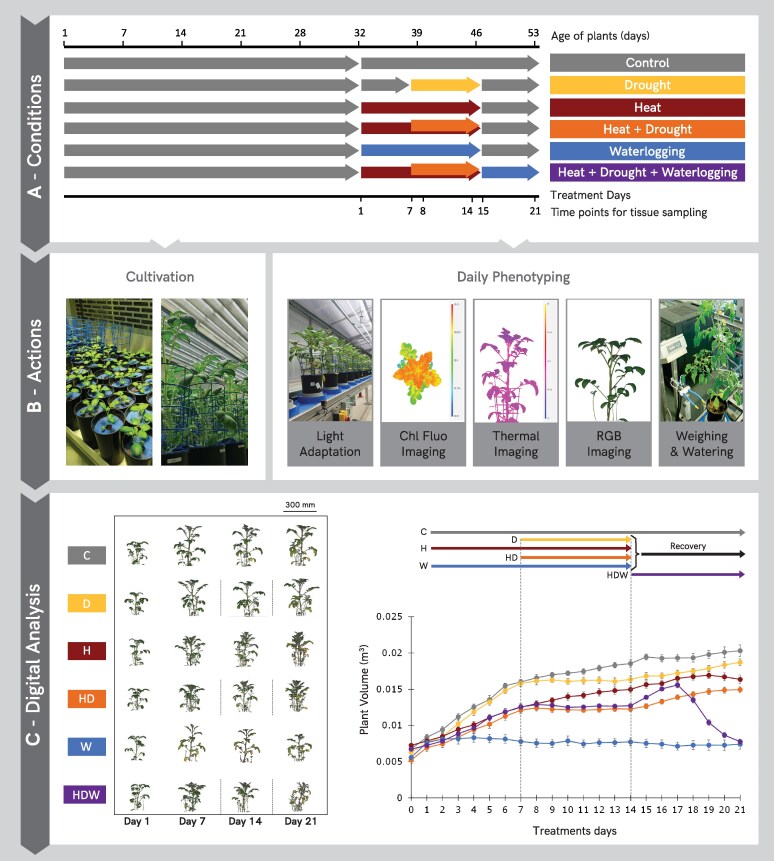
Overview of the experimental design for single- and combined stress treatments and multi-omics sampling. **A)** Summary of cultivation conditions. Timeline of the experimental set-up and applied stress treatments, including the recovery phase in potato cv. Désirée. Timing and duration of stress treatments and days for tissue sampling are shown. **B)** Actions comprised cultivation in the growing chambers and daily phenotyping with a set of sensors using the PlantScreen™ phenotyping platform at PSI Research Center. **C)** Automated image analysis pipeline was used to extract quantitative traits for morphological, physiological, and biochemical performance characterization of the plants during the stress treatment and recovery phase. Side view color segmented RGB images of plants were digitally extracted for comparison at selected time points of tissue sampling (left panel) and daily plant volume (m^3^) calculated from top and multiple angle side view RGB images (right panel). Black dotted lines reflect the initiation and removal of drought stress, respectively. Measurements, mean and standard error are shown (*n* = 6). C, control; D, individual drought stress; H, individual heat stress; HD, combined drought with heat stress; W, individual waterlogging stress; HDW, triple-stress condition.

This study aims to increase the mechanistic understanding of potato acclimation to individual and combined abiotic stresses. We focused on individual heat, drought, and waterlogging stresses, as well as realistic combinations of these. We used the cv. Désirée, a widely used moderately stress-resistant potato cultivar, as our model. To provide insights into multi-level regulation of stress responses, we conducted HTP and comprehensive omics analyses, according to the scheme outlined in Fig. 1A (for more details, see Supplementary Table S1).

### Effects of single and combined stresses on potato growth and morphology

To assess potato phenotypic responses to different stress conditions, multiple morphological and physiological traits (Supplementary Table S2) were quantified daily, using several imaging sensors (Fig. 1B). Using RGB side and top view imaging, we monitored changes in plant growth dynamics during the stress treatments and recovery phases, considering traits such as plant volume, area, height and compactness. We observed that all stress treatments negatively affected plant growth, however, to different degrees (Fig. 1C, Supplementary Fig. S1). Although individual drought (D, 30% of field capacity) and heat stress (H, 30 °C during the day, 28 °C at night) decreased the rate of biomass accumulation (plant volume, area, and height), we saw that heat had stronger effects over time (Fig. 1C, Supplementary Fig. S1, A to C). The negative effects of heat became more severe when combined with drought (HD, water withdrawal starting after 7 days of H) (Fig. 1C and Supplementary Fig. S1, A to C). Under HD, plants phenotypically resembled more heat-stressed plants, e.g. with respect to top area and compactness, however, with a clearly more negative effect (Supplementary Fig. S1, B and D). While heat stress caused hyponastic movement of leaves, waterlogging led to an epinastic leaf movement, which was accompanied by growth arrest and significant decrease in the top area, compactness and relative growth rate (RGR) that were observed after 1 day (Fig. 1C, Supplementary Fig. S1, A, B, D, and E, Table S3). In the 3rd week, when the single and HD stress treatments were finished (at treatment day 15), plants recovered well from D, H, and HD, as reflected by resumption of growth. This trend was not observed for plants subjected to W stress (Fig. 1C).

Plant performance was the worst in the triple-stress condition (HDW), where 7 days of H were followed by 7 days of combined HD and 7 days of W. Interestingly, during the first 3 days of W, that followed the period of heat and drought, plants grew very fast, but with a prolonged stress exposure, plants collapsed, as indicated by RGB side and top view images, plant volume and growth dynamics as shown in RGR and other measured morphological traits (Fig. 1C, Supplementary Fig. S1, Table S3).

### Effect of single and combined stresses on potato physiology

**Figure 2.**
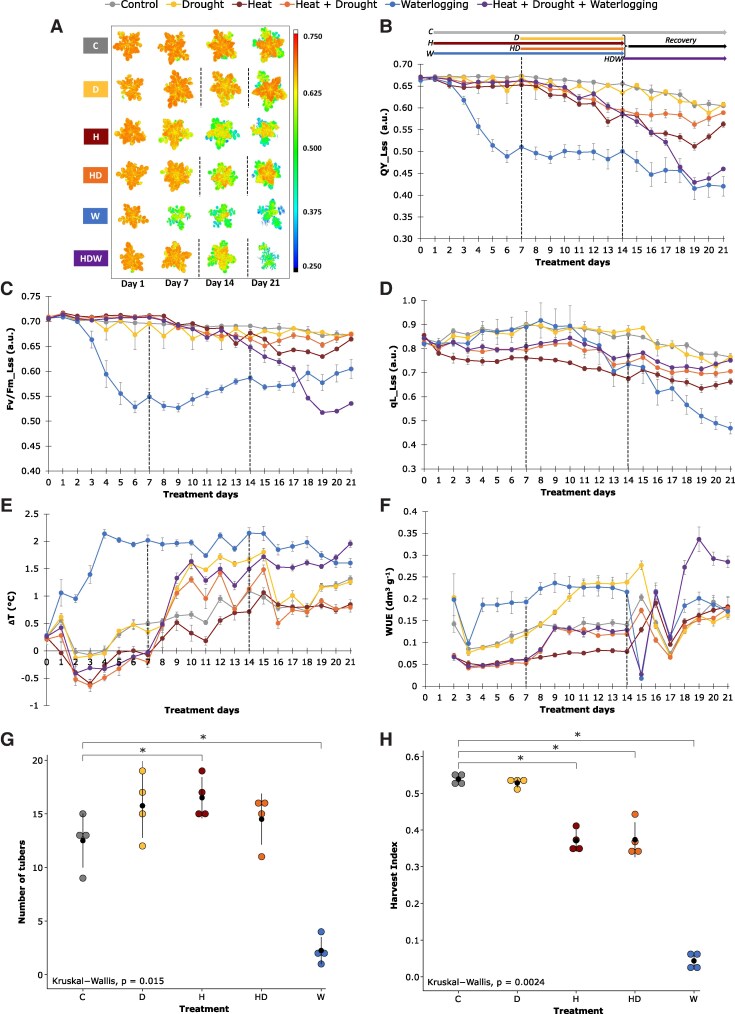
Physiological profiling using high-throughput phenotyping platforms reveals distinct responses to single and combined stresses. **A)** Pixel-by-pixel false color images of operating efficiency of photosystem II in light steady state (QY_Lss, arbitrary unit) captured by kinetic chlorophyll fluorescence measurement. Color scale bar represents the range of fluorescence values. Images for selected time points of tissue sampling were digitally extracted for comparison. Color coding of the treatments apply for the entire figure. Vertical dashed lines indicate the onset and end of drought. **B)** QY_Lss values extracted from images for each individual time point. **C)** Steady-state fluorescence of maximum efficiency of PSII photochemistry in the light trait based on chlorophyll fluorescence top view (F_v_/F_m__Lss). **D)** Steady-state estimation of the fraction of open reaction centres in PSII trait in light based on chlorophyll fluorescence top view (qL_Lss). **E)** Difference between canopy average temperature extracted from thermal IR images and air temperature measured in the thermal IR imaging unit (ΔT). **F)** Water use efficiency (WUE) based on plant volume and water consumption. **A-F)** Black dotted lines reflect the initiation and removal of drought stress, respectively. Measurements, mean and standard error are shown (*n* = 6). See Supplementary Table S3 for Statistical evaluation of differences between groups using Wilcoxon test. **G)** Tuber numbers counted per plant on the last day of the experiment (Day 28 = 60 days of cultivation). **H)** Harvest index calculated from the total biomass and tuber weight on the last day of the experiment. **G** and **H)** Measurements, mean and standard deviation are shown (*n* = 4). Statistical evaluation of differences between groups is given by the non-parametric Kruskal–Wallis test (1-way ANOVA on ranks); *P*-value above *x* axis, where asterisk denotes *P*-value < 0.05. See Fig. 1A for scheme on stress treatments. C, control; D, individual drought stress; H, individual heat stress; HD, combined drought with heat stress; W, individual waterlogging stress; HDW, triple-stress condition.

To evaluate photosynthetic performance under single and multiple stresses, a broad range of physiological traits were extracted from chlorophyll fluorescence images and analysed (Fig. 2). Top view images of the operating efficiency of photosystem II (PSII) in light steady state (QY_Lss) clearly showed the negative impact of stress on photosynthetic capacity in all stress treatments, indicated by the reduction of QY_Lss, with D stress causing only a weak negative effect (Fig. 2, A and B). Moreover, steady-state fluorescence of maximum efficiency of PSII in the light (F_v_/F_m__Lss) showed (only) a significant decrease after 3 days in W alone and when W followed a period of HD till the end of the experiment, indicating a high stress level (Fig. 2C, Supplementary Table S3). A decrease in steady-state estimation of the fraction of open reaction centers in PSII in the light (qL_Lss) was observed after 1 day of H and remained consistently lower than in other conditions. In contrast, D had no significant effect on these parameters (Fig. 2D, Supplementary Table S3). When drought was combined with heat stress (HD), an increase in qL_Lss as compared with H alone was observed. Following H and HD stress, qL_Lss values did not recover back to the control levels at day 21 (Fig. 2D), suggesting that photosynthesis is enduringly affected. There was a slow decrease in qL_Lss after long-term W with a clear decline after stress recovery (Fig. 2D). In addition, changes in canopy temperature (ΔT) were deduced from the thermal imaging, while water use efficiency (WUE) was calculated based on plant volume and water consumption. The rapid increase in ΔT and WUE under W and HDW was most likely caused by rapid stomatal closure (Fig. 2, E and F). The strong response remained over the entire stress period, and plants were unable to recover from both stress treatments. A steady increase in ΔT and WUE was observed starting at 3 days in D, suggesting that the stress was recognized, and the plants responded by closing stomata. When D stress was removed on day 15, the plants recovered immediately (Fig. 2, E and F). Heat stressed plants showed a decrease in ΔT together with an increase in water consumption and a lower WUE, thus indicating enhanced leaf cooling (Fig. 2, E and F, Supplementary Tables S3 and S4). During combined HD stress, an intermediate response was observed for these physiological traits compared with single D and H stress.

### Stress combinations and waterlogging have strong effects on potato yield

At the end of the phenotyping, plants were harvested to assess the total biomass accumulation and tuber yield (Fig. 2, G and H). Single H stress led to a slightly higher tuber number (Fig. 2G). However, compared with control conditions, tubers were smaller and weighed less resulting in a lower harvest index. HD also significantly reduced the harvest index compared with control conditions, while D alone did not affect final tuber yield (Fig. 2H). W stress strongly inhibited tuber formation and growth and only a few tubers were formed, leading to a significant reduction in the harvest index compared with the control treatment (Fig. 2H). A combination of all stress factors abolished tuber formation, reflecting the (near) lethal effect of HDW (Fig. 2, G and H).

Negative effects of the stress treatments on tubers were also observed at the metabolic level (Supplementary Fig. S2). Thus, starch content was significantly lower under H, HD, and W stress, while D stress alone had no negative impact. The accumulation of hexoses under H and HD may hint to an increased starch degradation and/or to a reduced starch biosynthesis. W caused a strong accumulation of almost all amino acids, most likely caused by protein degradation and a hampered metabolism (Supplementary Fig. S2).

### Molecular responses across omics levels reveal mechanistic insights into multi-stress acclimation

**Figure 3.**
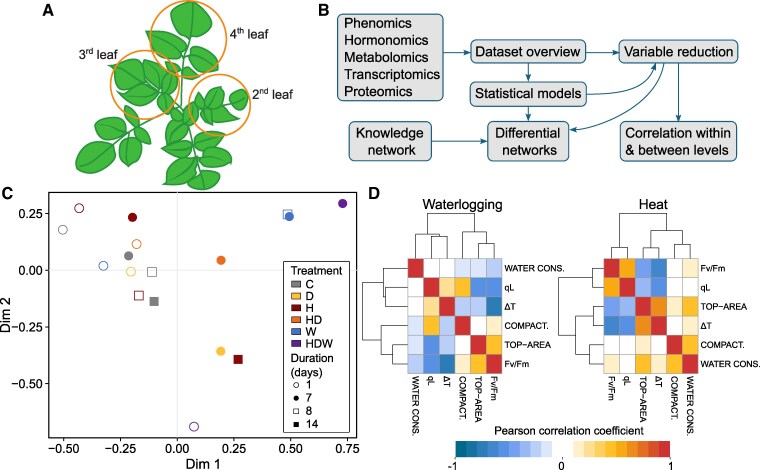
Integrated analysis of measured and generated data permits global visualization and multi-level amalgamation of potato stress responses. **A)** Schematics of tissue sampling protocol. 2nd and 3rd leaves were harvested for destructive “omics” analysis, 4th leaf was used for relative water content calculation. Remaining plant tissue was quantified to obtain total above-ground biomass and tuber yield. **B)** Overview of data analysis pipeline. **C)** Dataset overview: multidimensional scaling shows combined HDW stressed plants as extremes, the centroid of each plant group is shown. **D)** Most informative variables from the phenomics level. Pearson correlation coefficients between them are presented as hierarchically clustered heat maps in waterlogging and heat stress. Abbreviations—Fv/Fm: F_v_/F_m__Lss, qL: qL_Lss, top-area: top area, compact: compactness, water cons.: water consumption. For trait description, see Supplementary Table S2.

In addition to the morphological and physiological measurements (68 variables, Fig. 3D, Supplementary Tables S1 and S2), leaf samples were taken for parallel multi-omics analysis. The second and third mature leaf per plant were pooled, homogenized, and used for further analysis (Fig. 3A). For each of the treatments the fast response (1 day post-treatment) and the status at the end of a prolonged stress duration (7 or 14 days of stress) was investigated (sampling points see Fig. 1A). While the proteome analysis was untargeted (4258 identified proteins, Supplementary Tables S5 and S6), other omics analyses were targeted comprising 14 pre-selected transcriptional marker genes involved in stress response and tuberization, 13 phytohormones encompassing abscisic acid, ABA; jasmonic acid, JA; salicylic acid, SA; indole-3-acetic acid IAA, and their derivatives as well as 22 metabolites encompassing amino acids and sugars (Supplementary Table S4). To identify processes regulated on proteomics level, we performed gene set enrichment analysis (GSEA, Supplementary Table S6).

A multi-level data integration protocol was developed to investigate plant signaling/responses across the different omics levels (Fig. 3B). First, we investigated data distribution by multidimensional scaling (Fig. 3C, Supplementary File 1). This graph shows a clear clustering aside of samples taken after 7 and 8 days of waterlogging. Therefore, only data from the first week of waterlogging were included in further analyses, taking also into consideration that after 2 weeks of waterlogging all plants were severely damaged. The overview of data distribution also revealed that the most distant physiological state was that of plants exposed to triple stress (HDW): (Fig. 3C). Because the triple stress treatment turned out to be very harsh and plants were severely affected in both above ground and below ground growth, we also excluded data from these samples from all further analyses. Next, we reduced the number of variables obtained on phenomics and proteomics levels to equalize numbers of variables across different analyzed levels. In order to identify the most informative variables, feature selection using random forest with recursive feature elimination was conducted on the phenomics data, keeping 6 variables for downstream analysis (Fig. 3D: qL_Lss, F_v_/F_m__Lss, top area, ΔT, compactness and water consumption). The proteomics dataset was reduced to keep only proteins that were identified as differentially abundant in any comparison of stress vs. control (135 proteins) and were functionally assigned to pathways that were studied also on other levels (36 proteins, related to photosynthesis, metabolism of sugars and amino acids, hormone metabolism and signaling, ROS signaling, and stress pathways).

In addition, correlation analysis within each level of omics data was performed, revealing that these components are only weakly connected in control conditions, while in both heat or drought, they are highly correlated to each other (see e.g. for hormones and transcripts, Supplementary Fig. S3, A and B). More severe stresses, such as the combined heat and drought stress and waterlogging, however, broke this link, suggesting a disorganization of signaling responses. The canonical correlation analysis between components of different molecular levels similarly showed low connection in control samples. In stressed samples, blocks of components appeared to be strongly regulated, each specific to a particular stress (Supplementary Fig. S3C).

**Figure 4.**
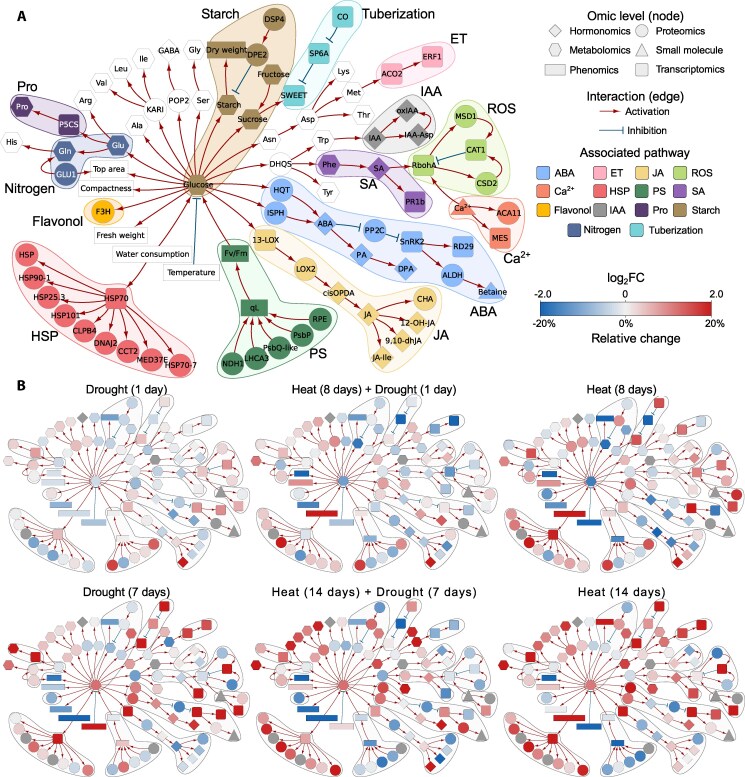
Integration of multi-omics data in a knowledge-based metabolic and signaling network. **A)** Structure of knowledge network. Individual studied components are colored according to their function in different pathways. **B)** To compare the effects of different stresses on the overall state of the plant, we overlaid the knowledge networks with measured changes in component concentration. Nodes are colored by log2 fold changes (red—increase in stress compared to control, blue—decrease in stress compared with control, grey—measurement not available) shown for 2 time points: sampling day 8 and sampling day 14 for the different stress treatments, days of stress treatment are given with each network (for more details of the set up see Fig. 1A). Displayed omics measurements were obtained from leaf samples. Identifiers and descriptions corresponding to the short names shown in graphs are available in Supplementary Tables S3 and S5. ABA, abscisic acid; Ca2+, Calcium; ET, ethylene; HSP, heat shock protein; IAA, indole-3-acetic acid (Auxin); JA, jasmonic acid; Pro, proline; PS, photosynthesis; ROS, reactive oxygen species; SA, salicylic acid.

Variables measured on different omics levels were integrated into a metabolism and signaling cascade-based knowledge network to capture events at the molecular level (Fig. 4A). Finally, we superimposed the measured data onto this mechanistic knowledge network and visualized them in parallel for all omics levels per each analyzed condition compared to control (Fig. 4B, Supplementary Table S3, File 2). This provides a comprehensive overview of how these stresses rewire biochemical pathways and physiological processes. These networks were used for interpretation of processes in single and combined H and D stress as well as for W and are described in the subsequent sections.

### Metabolic and molecular responses to individual and combined heat- and drought stress exhibit combinatorial and distinct signatures

While heat stress was effective immediately, drought stress, applied by water withdrawal on day 7 in our setup, became effective gradually within 3 days, visible by an increase in the ΔT values (Fig. 2E). Like previous reports (Demirel et al. 2020; Zaki and Radwan 2022), we found that Désirée was moderately drought tolerant and exhibited only minor morphological and physiological responses at the moderate stress level applied in our study (30% field capacity) and the plants fully recovered when the stress treatment was finished. The potato plants clearly responded to elevated temperatures (H) with morphological adaptation like the upward movement of leaves (Fig. 1C), which is part of thermomorphogenic responses (Quint et al. 2016). Previous work showed that heat stress caused an altered biomass allocation between shoots and tubers of potato plants, with less assimilates allocated to developing tubers (Hancock et al. 2014; Hastilestari et al. 2018). Decreased tuber yield (higher number of tubers with smaller biomass, Fig. 2G) and starch accumulation, leading to a lower harvest index, were also observed in our study (Fig. 2H and Supplementary Fig. S2).

**Figure 5.**
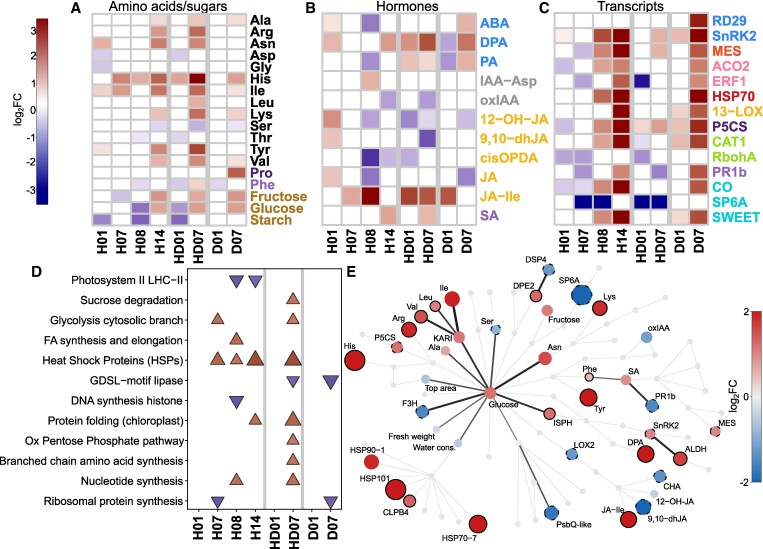
Combined heat and drought stress trigger distinct responses compared to each individual response. Additive effect of combined stress is most pronounced for branched chain amino acids accumulation and JA signaling response. **A–C)** Heatmaps showing log2FC (FDR *P*-value < 0.05) in individual stress heat (H) or drought (D) stress in comparison to combined one (HD) for targeted molecular analyses. Label colors indicate pathway associated with each molecule as in the Knowledge network (see Fig. 4 for legend). **A)** Changes in metabolite levels. **B)** Changes in hormone levels, and **C)** Changes in selected stress-related transcripts. **D)** Changes observed on proteomics level. Results of Gene Set Enrichment Analysis (FDR q-value < 0.1) are shown. For more information see Supplementary Table S6. **E)** Biochemical knowledge network showing changes under combined HD stress at day 14 (treatment day 7). In this version of knowledge network, only nodes that were significantly differentially expressed (vs. control conditions) are colored and the connections between 2 differentially expressed nodes are colored black. Node full black border indicates molecules with higher expression levels in HD compared with H and/or D alone. Dashed black border indicates molecules with lower expression levels in HD compared with H and/or D alone (difference of log2FC > 0.5). Displayed omics measurements were obtained from leaf samples. Identifiers and descriptions corresponding to the short names shown in graphs are available in Supplementary Tables S3 and S5.

To investigate the effect of heat and/or drought stress on leaf carbohydrate metabolism, contents of soluble sugars and starch were measured (Fig. 5A). While sucrose levels did not change (Supplementary Table S3), there was an about twofold increase in the amount of fructose and glucose after 14 days of H and at day 7 of D and HD. Under H and HD combination also less starch accumulated in leaves (Fig. 5A). This most likely reflects the decreased photosynthetic assimilate production and contributes to a lower amount of carbon that can be transported to sink organs, such as growing tubers to stimulate growth and starch deposition. The soluble sugars may act as osmoprotectants under these stress conditions, feed the increased demand for energy and serve as building blocks for stress defense responses. Accordingly, we found that, enzymes involved in glycolysis or sucrose degradation were upregulated in H and combined HD stress as indicated by gene set enrichment analysis, which summarizes the complex proteomics data set (Fig. 5D and Supplementary Table S6).

Expression of the sugar efflux transporter *SWEET11* was upregulated at the end of both H and D stress presumably to maintain sucrose loading into the phloem and carbon allocation to sink tissue to counterbalance the decreased carbon assimilation rate of source leaves. This view is supported by various other studies demonstrating an emerging role of SWEET sugar transporters in abiotic stress responses as summarized in Gautam et al. (2022). For example, Chen et al. (2022) showed that *AtSWEET11/12* are rapidly activated under drought stress in an ABA and SNF1-related protein kinase 2.9 (Snrk2)-dependent manner to enhance assimilate allocation from shoot to root to stimulate root growth and allow stress adaptation. One key player stimulating tuberization and tuber growth is the tuberigen *SP6A*. Its expression was downregulated during the first week of H and in combined HD (Fig. 5C). During longer heat exposure, expression levels of *SP6A* were similar to control levels, but remained low in HD. Drought alone had little effect on *SP6A*, which is consistent with the low impact on final tuber yield. In potato, the transcriptional regulator protein *Constans-like 1* (CO) was described to act as a negative regulator of *SP6A* expression (Abelenda et al. 2016). *CO* was upregulated within 7 days of drought stress (D), and it increased with longer durations of heat, but was unaffected by HD combination (Fig. 5C). Hence, in our experiment, the transcript levels of *CO* did not always change in the opposite direction as *SP6A*, suggesting additional regulatory mechanisms may act under stress conditions.

Considering the changes in amino acids, the most striking finding was the strongly elevated levels of histidine (His) in all 3 stress treatments, with the highest amounts detected in combined HD stress (Fig. 5A). This was accompanied by a significant increase of many (minor) amino acids, in particular isoleucine (Ile) and other branched chain amino acids (BCAs). This observation was in line with previous reports on combined heat- and drought stress in potato (Demirel et al. 2020), although its cause and physiological importance need further investigations. Accordingly, at the proteome level, proteins involved in BCA synthesis were significantly enriched among the ones with increased levels in stress (Fig. 5, D and E, Supplementary Tables S5 and S6).

Proline is an established regulator of osmotic potential that protects cells by stabilizing proteins and scavenging ROS. Proline levels increased at day 7 of D stress (sampling day 14; Fig. 5A). This was consistent with increased transcript levels of the *delta-1-pyrroline-5-carboxylate synthase 1* (*P5CS*), the key enzyme for proline synthesis, and of *Responsive to Desiccation 29B (RD29B*), both being well-known stress marker genes. In line with these findings, the levels of ABA, the key phytohormone that induces stomal closing, proline accumulation and other drought stress responses (Cutler et al. 2010; Zhang et al. 2022a, 2022b), were elevated after 7 days of D but were clearly reduced in H, while no changes were detected in HD stress. Interestingly, the levels of phaseic acid (PA), and dihydrophaseic acid (DPA), 2 breakdown products of ABA, were lower at 1 day of D but significantly higher after 7 days in D. The strongest accumulation of DPA levels was detected in the HD treatment, in which DPA levels were elevated already after 1 day and further increased until the end of the treatment (at day 7). The elevated levels after 1 day of HD can be explained by the experimental setup, in which the D treatment started after 7 days of H (that also resulted in DPA accumulation). However, the strong accumulation of ABA breakdown products under D and to even higher levels in HD are in line with their suggested role in long-term stress acclimation. PA, the first degradation product of ABA and precursor of DPA, is known to activate a subset of ABA receptors (Weng et al. 2016). Because ABA has a very short half-life, it was suggested that the long-lived PA could prime plants for enhanced responses to future drought (Lozano-Juste and Cutler 2016).

The phytohormone JA is another typical stress hormone known to be involved in many biotic but also abiotic stress responses (Wasternack and Feussner 2018). We found strongly increased levels of the biologically active form jasmonyl-L-isoleucine (JA-Ile) under H, D, and HD conditions (Fig. 5B). 12-Hydroxyjasmonic acid (12-OH-JA) is a by-product of switching off JA signaling with weak signaling activity (Nakamura et al. 2011). It was also described to function as tuber-inducing factor in potato (Yoshihara et al. 1989). Under H, amounts of 12-OH-JA and free JA switched from higher amounts measured at day 1 to lower levels at day 8 and decreased further till the end of the experiment. Also, *cis*-12-oxo-phytodienoic acid (*cis*-OPDA), the biochemical precursor of JA, was detected at much lower levels on day 8 and 14 in H. Cis-OPDA was also reduced at the start of the combined HD (day 1) treatment, most likely because of prior heat treatment. Altogether, this indicates a strong upregulation in the last step of conjugation for the synthesis of JA-Ile in H, D and HD.

The accumulation of ROS is a detrimental by-product of photosynthesis and other metabolic pathways under stress conditions. Accordingly, ROS detoxification by different enzymes such as catalases or superoxide dismutases, together with induction of Ca^2+^ signals, is a typical response emerging from stressed chloroplasts (Stael et al. 2015). In line with that, we measured increased transcript levels of *CATALASE 1* (*CAT1*) and a methyl esterase (*MES*), which was selected as Ca^2+^ signaling marker gene (M. Knight, unpublished data), at the end of the drought and heat treatment (sampling day 14). The transcript levels of *pathogenesis-related protein 1b1* (*PR1b*), a biotic stress as well as drought and salt stress marker (Akbudak et al. 2020) were first lower in H but increased from day 8 to 14 in H and at day 7 in D (Fig. 5C). A similar response was seen for the chloroplast-localized *13-lipoxygenase (St 13-LOX3.1)*, which is a well-known marker gene for different stresses, especially chloroplast generated ROS (Bachmann et al. 2002) suggesting increased ROS formation and stress level with stress duration. Strikingly, the expression of these genes was less induced or even inhibited when H and D were combined, which may indicate the activation of opposing signaling pathways.

Heat stress, but also other stresses, induces the production of heat-shock proteins (HSPs), which is a very conserved process in all organisms. HSPs act as molecular chaperones and play an important role in maintaining cellular homeostasis and the proteome by supporting protein folding, preventing misfolding or by assisting in the degradation of irreversibly damaged polypeptides (Sato et al. 2024). At the transcript level, we observed clearly elevated levels of the *heat shock protein 70* (*HSP70*) after single H and D stress. Under H this was accompanied by an accumulation of numerous HSP proteins as indicated by their significant enrichment among the identified proteins in the proteome approach (Fig. 5, D and E). This effect was similarly pronounced in combined HD stress (Fig. 5E) with a strong enrichment of HSP70, HSP90, and HSP101 involved in heat stress responses and protein folding (Fig. 5D). The category “protein folding” comprises mainly HSP70 and 60 group members many of which are present in the chloroplast, where they participate in the repair of PSII components, but also protect enzymes such as Ribulose-1,5-bisphosphate carboxylase/oxygenase (RuBisCo). In fact, our physiological data indicate a disturbance in the electron-transport chain through PSII under heat. For example, we saw a strong decrease of PSII efficiency under H stress (Fig. 2B). The negative effect of H stress is also reflected in lower abundance of PSII proteins in the gene enrichment analysis (Fig. 5D). More specifically (Supplementary Table S5), there were reduced amounts of the PsbQ and PsbP subunits of the oxygen-evolving complex.

Overall, we do see specific stress responses to heat and drought but also to a combination of both. This is evident in Fig. 5E illustrating the signature of responses elicited by combined heat and drought stress in biochemical pathway view (knowledge network overlaid with multi-omics data). The responses to HD only partly overlap with single D stress, e.g. for the accumulation of DPA. However, for most of the measured metabolites, the patterns were similar to those observed under heat stress, with changes being more pronounced under HD (Fig. 5E). Here, we cannot exclude that this domination by heat was linked to the rather mild drought stress. Interestingly, the transcriptional changes of selected stress-related enzymes were the weakest in HD stress combination pointing to a redirection and rearrangement of signaling pathways compared with individual stress factors as suggested by other studies (Zhang and Sonnewald 2017).

### Comprehensive insight into molecular processes mediating the extreme waterlogging sensitivity of potato

Despite being documented as a highly flood-sensitive species, an in-depth characterization of flooding-induced stress responses in potato is sparse (Jovović et al. 2021). The waterlogging sensitivity of potato was evident in the HTP data, with several morpho-physiological traits related to plant performance being negatively impacted following stress imposition (Figs. 1C and 2). These included leaf epinasty, decreased biomass accumulation and shoot elongation, impaired photosynthesis and stomatal conductance as well as a dramatic reduction of tuber yield (see Figs. 1 and 2).

**Figure 6.**
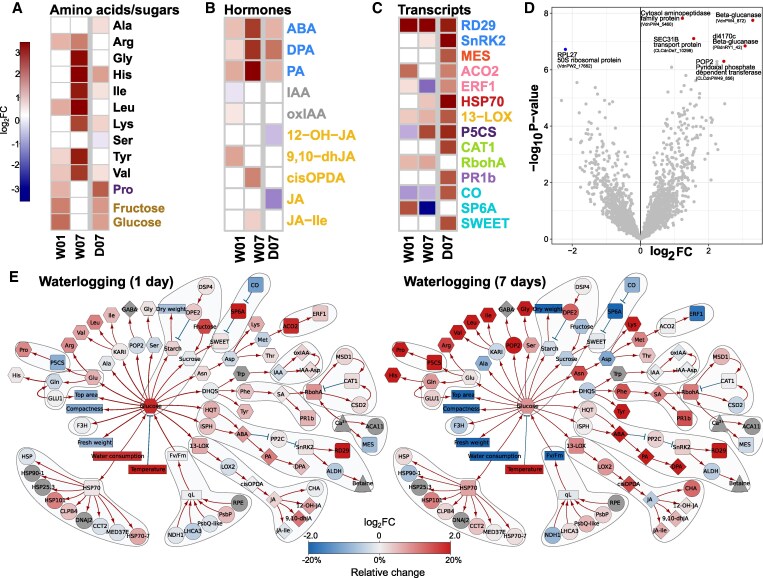
Waterlogging triggers drought-stress like molecular responses in potato. **A-D)** Heatmaps showing log2FC (FDR *P*-value < 0.05) changes in **A)** metabolite levels, **B)** phytohormones, **C)** selected stress-related transcripts. **D)** Volcano plot of differential proteomics analysis at day 7. Proteins with FDR *P*-value < 0.05 shown as blue (downregulated) and red (upregulated) dots. For more information, see Supplementary Table S5. **E)** Knowledge network of waterlogging stress at day 1 and day 7 (unfiltered, color range [−2, 2]). For legend, see Fig. 4. Displayed omics measurements were obtained from leaf samples. Identifiers and descriptions corresponding to the short names shown in graphs are available in Supplementary Tables S3 and S5. D, individual drought stress; W, individual waterlogging stress.

Waterlogging significantly affected primary metabolic pathways as reflected in an increase in soluble sugars and free amino acids (Fig. 6A). We also observed changes in the expression of stress-associated genes and hormones, thus highlighting potential mechanisms involved in waterlogging acclimation. These include the increase of ABA (ABA, PA, DPA) metabolism and response (*RD29B),* as well as the upregulation of the ethylene (ET) biosynthesis gene, 1-aminocyclopropane-1-carboxylate oxidase 2 (*ACO2),* and the ROS-producing enzyme, respiratory burst oxidase homolog A (*RBOHA).* Waterlogging also resulted in the accumulation of various JA metabolites, 9,10-dihydrojasmonic acid (9,10-dHJA), cisOPDA, and JA-Ile, along with the upregulation of *13-LOX3.1*, an enzyme involved in JA metabolism. The strong ABA signature observed in waterlogged plants prompted us to compare waterlogging and drought responses (Fig. 6B). This revealed common stress-associated responses (e.g. the induction of *ACO2*, *RD29B, HSP70*) and a much stronger ABA response in waterlogging relative to drought. Another notable observation was the upregulation of the tuberigen signal, *SP6A* after 1 day of waterlogging, coinciding with the downregulation of its negative regulator *CO* (Fig. 6C).

Proteomics analyses of waterlogged plants revealed mild effects. Six differentially enriched proteins were identified in response to prolonged (7 days waterlogging, W07) waterlogging treatment. Among the strongly upregulated proteins were a leucyl aminopeptidase 2-like (LAP2-like) (VdnPW4_5460), 2 glucan endo-1,3-beta-glucosidases (VdnPW4_8729, PBdnRY1_427), a Pollen-Pistil Incompatibility 2-like protein (POP2-like) encoding a gamma-aminobutyric acid (GABA) transaminase and a component of the coat protein complex II, SECRETORY31B-like protein (SEC31B-like), involved in vesicular transport from the endoplasmic reticulum (ER) to the Golgi apparatus (Li et al. 2021). Waterlogging led to the downregulation of the chloroplast RIBOSOMAL PROTEIN L27A-like (RPL27-like), demonstrated to be important for protein synthesis (Fig. 6D).

The multi-level integrative analyses enabled visualization of the progression of stress symptoms in waterlogged plants. In comparison to 1 day of waterlogging, molecular responses to prolonged waterlogging stress displayed a distinct signature (Fig. 6E). While ABA-, JA- and ROS-biosynthesis and accumulation of free amino acids were further increased, we observed that prolonged waterlogging led to a general inhibition of the tuberization process (i.e. *SP6A* downregulation). In addition, genes related to ethylene biosynthesis (*ACO2)* and response (ETHYLENE RESPONSE FACTOR1, *ERF1)* were no longer found to be transcriptionally upregulated, thus suggesting temporal control of ethylene signaling. Despite representing opposite ends of the water stress spectrum, waterlogging and drought, elicited significantly overlapping responses, notably related to ABA metabolism and proline accumulation (Fig. 6, A to E).

## Discussion

Despite its outstanding importance as a major food crop, research into the vulnerability of potato to abiotic stresses lags that of other staple crops. In recent years, potato yields have been significantly affected by heat, drought, and flooding, often occurring sequentially or simultaneously (Dahal et al. 2019; Jovović et al. 2021; von Gehren et al. 2023). Considering the increasing occurrence of these extreme weather events, this knowledge gap needs to be urgently addressed. In this study, we leveraged the power of several omics techniques and their integrated analysis to build a comprehensive global picture of potato responses to single and combined heat, drought, and waterlogging stress.

### Leveraging multi-omics data integration to capture the complexity of biological systems

Several tools have already been developed for integrative analysis of multi-omics data (Joshi et al. 2024). Most broadly used are the mixOmics package (Rohart et al. 2017; Singh et al. 2019), integrating datasets based on correlations, and the pathway visualization tool PaintOmics (Liu et al. 2022). In this study, however, we integrated 5 omics-level datasets. Such complex datasets have rarely been analyzed, even in medical research (Lee et al. 2019), as most studies combine only 2 to 3 omics datasets (Ployet et al. 2019; Lozano-Elena et al. 2022; Núñez-Lillo et al. 2023, 2024; Sinha et al. 2024). Since existing tools were not directly suitable for our needs, we developed a pipeline harnessing the potential of both integrative and visualization approaches. An additional step, based on machine learning, was introduced to reduce the number of variables, in particular of phenotypic physiological data. This reduction of variables was especially important for the correlation analyses across omics levels, where we kept only the most informative variables. In the first step, we performed statistical modelling and correlation analyses, which provided partial overview of events. In the second step, mechanistic insights were obtained by generating a customized biochemical knowledge network. Our network was constructed based on knowledge extracted from different databases, most notably the Stress Knowledge Map (Bleker et al. 2024) and KEGG (Kanehisa et al. 2017), as well as from literature, to integrate all components that were kept after variable selection. The obtained biochemical knowledge network enabled a comprehensive overview of events at the pathway level and led to the identification of mechanistic differences occurring in response to different stresses. The developed pipeline is thus highly useful for integration and interpretation of complex datasets in future studies and can also be applied to other species.

### Integrative omics provides global insights into potato abiotic stress responses

Multi-omics approaches have been successfully applied in numerous crop species to better understand abiotic stress responses. Our study does so in potato by subjecting the cultivar Désirée to waterlogging, drought, heat, a combination of heat and drought, and triple stress combination encompassing all 3. Across each stress treatment, detailed morpho-physiological traits were measured, with a subset of plants sampled for the probing of a diverse array of molecular stress markers, hormones, metabolites, and proteome analyses across several time points.

**Figure 7.**
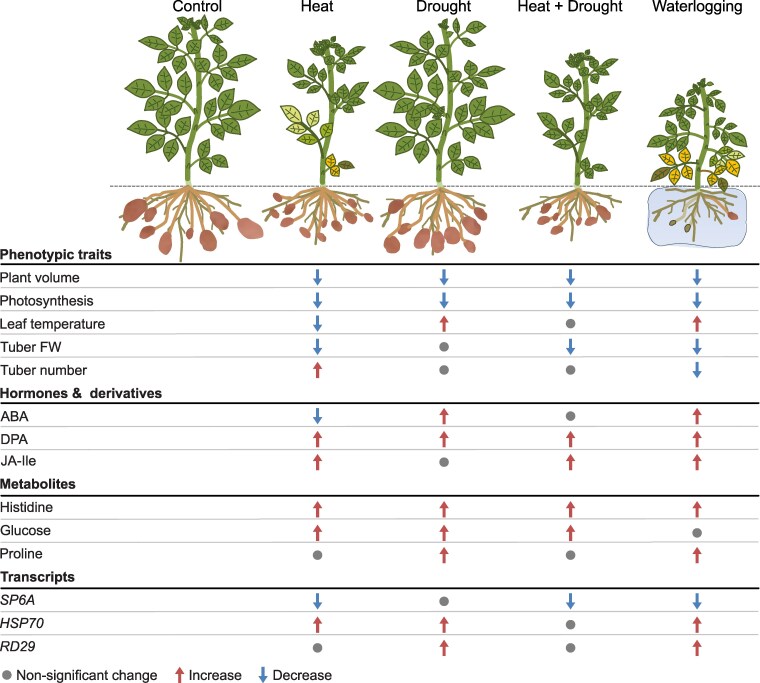
Schematic summary of multilevel responses to single and combined heat, drought, and waterlogging stresses. Selected variables from each level are shown. Summary of molecular responses (hormones, metabolites, and transcripts) was based on the comparisons illustrated in Figs. 5 and 6. Summary of morphophysiological responses were based on the data from the last day of the experiment (Day 28), which includes tuber information. Proteomics data set is not included here due to the small dataset of differentially expressed proteins in the waterlogging treatment. Degree of increase or decrease is not specified. ABA, abscisic acid; DPA, dihydrophaseic acid; Ja-Ile, Jasmonoyl-isoleucine; *SP6A*, *SELF-PRUNING 6A*; *HSP70*, *heat shock protein 70*; *RD29*, *responsive to desiccation 29B*.

In general, stress combinations appeared to be more detrimental to the plant performance than individual stress applications. The combination of H, D, and W led to a rapid decline in plant viability and eventually most plants died. However, all individual stress factors caused a reduced plant growth, had a negative impact on photosynthetic assimilate production, and both heat and waterlogging stress impaired tuber yield (Fig. 7) and tuber starch accumulation (Supplementary Fig. S2). Considering all stress responses, it turned out that the cultivar Désirée was less affected by the applied drought stress indicating that it is quite resilient to drought as suggested previously (Demirel et al. 2020). A combination of heat and drought caused stronger growth retardation than both stresses alone with drought responses overwriting heat adaptations.

Nevertheless, despite the apparent mild drought phenotypes, a clear drought-associated signature (accumulation of ABA, sugars, proline, histidine and most stress-induced transcripts) confirmed the activation of stress responsive pathways, particularly at day 7, which contributed to stress acclimation (Fig. 5). Thus, there was a clear activation of the ABA response pathway, as seen by an increase in the content of the hormone and its degradation products, as well as proline, and the increased expression of ABA-responsive marker genes *SnRK2*, *P5CS*, *RD29B*, leading to the corresponding physiological responses, e.g. a decreased water-use and leaf temperature caused by stomata closure. Particularly interesting was the accumulation of the ABA catabolite DPA after longer drought stress. The precursor of DPA, PA was suggested to have an important role in priming for increased resilience to future drought stress in Arabidopsis (Lozano-Juste and Cutler 2016). It is believed that DPA does not trigger ABA responses but that has, to our knowledge, not been studied in potato. Hence, it might be that DPA acts as priming signal for stress acclimation and resilience in potato.

Compared with drought, heat stress had a stronger impact on Désirée plants at all levels from growth to photosynthesis and yield. Thermomorphogenesis is a well-described morphological response to elevated temperature stress comprising shoot elongation and hyponastic movement of leaves which together with an increased transpiration are seen as an acclimation to increase ventilation and to cool the aboveground part (Quint et al. 2016). In our study, we clearly observed the hyponastic movement of leaves and stomatal opening, indicated by a decrease in ΔT (Fig. 7). This physiological response was accompanied by a decreased amount of ABA. In contrast, the heat-mediated shoot elongation that has been seen in other potato varieties was not visible in Désirée (Hastilestari et al. 2018; Tang et al. 2018). Instead, the plant height of Désirée plants was reduced under elevated temperature suggesting cultivar-specific difference that could be exploited in further studies to untangle different morphological stress adaptation mechanisms in potato. In *Arabidopsis thaliana*, the thermomorphogenetic hypocotyl elongation is tightly linked with an increase in auxin levels and is mediated by the transcription factor Phytochrome interacting factor 4 (PIF4) (Quint et al. 2016). Consistent with the morphological response, we did not find significantly altered levels of the phytohormone IAA in leaves of Désirée plants in response to heat (Fig. 5B).

High temperature treatment negatively affects photosynthetic capacity, particularly the efficiency of photosystem II, which is in line with results of other studies (Mathur et al. 2014), and this was confirmed here at both physiological and proteomic level. In a previous study by Hancock et al. (2014), which also used cv. Désirée, the net CO_2_ assimilation was even higher under elevated temperatures than in control conditions. This difference might be related to the different setup, as in the latter study the night temperature was kept at 20 °C, while here it was adjusted to 28 °C, suggesting that a low night temperature is important to maintain photosynthetic activity. The heat-induced impact on photosynthetic capacity most likely caused a lower production of assimilates, indicated by the decreased amount of transitory starch in leaves. Concomitantly, contents of hexoses were found to be increased consistent with earlier studies (Hastilestari et al. 2018). This increase may contribute to the osmoprotection of cells and provides energy for the costly heat stress response such as the formation of heat shock proteins (Guihur et al. 2022). A massive accumulation of heat shock proteins was found after 1 week of heat stress, together with elevated levels of *HSP70* transcript levels at day 8 (Fig. 5C). Although energy-demanding, the transcriptional induction of *HSP* is important for the cellular homeostasis and maintenance of growth and metabolism at elevated temperature. This is well demonstrated by transgenic potato plants with increased expression of a beneficial allele of *HSP70*, which exhibit improved heat stress tolerance (Trapero-Mozos et al. 2018).

A downregulation of photosynthesis is a typical stress response to prevent potential damage, for example, caused by ROS. This has strong implications on plant growth and yield and is therefore regulated at various levels including light-harvesting and electron transport with high implications for crop improvement (Kromdijk et al. 2016), particularly under stress conditions (Grieco et al. 2020). The resulting lower photosynthetic capacity together with an increased energy demand for stress defense reduces the amounts of assimilates that can be translocated toward the developing tubers and its availability for storing starch. In addition to assimilates, molecular signals play a critical role in stimulating tuber development and growth. One important regulator is *SP6A*, which was downregulated at the transcript level in our study, consistent with previous findings (Hancock et al. 2014; Lehretz et al. 2020; Park et al. 2022; Koch et al. 2024). The downregulation of *SP6A* likely contributes to the observed reduction in tuber yield under stress conditions. Notably, stem-specific overexpression has been shown to overcome heat-mediated yield reduction by enhancing delivery of assimilates to developing tubers, thereby improving tuber growth and starch accumulation.

Looking at plant hormones, we observed changes of stress hormones like SA and JA, which traditionally have been associated with biotic stress responses. Quite striking in this context was the increase in the amount of JA-Ile under heat, drought and the combination of both (Fig. 5B). This is consistent with previous observations reporting that JA has a positive effect on thermotolerance in Arabidopsis (Clarke et al. 2009; Balfagón et al. 2019). Heat stress increased levels of OPDA, JA, and JA-Ile and application of 5 *µ*M methyl-jasmonate improved cell viability (Clarke et al. 2009). Using various mutants, this study also showed that JA acts in concert with SA in conferring thermotolerance. Moreover, an accumulation of JA-Ile was also observed in Arabidopsis under drought (Yoshida and Fernie 2024), and increased levels of JA-Ile by overexpression of *JASMONATE RESISTANT1 (JAR1)* resulted in improved drought stress tolerance, but in stunted growth (Mahmud et al. 2022). Detailed analyses of how levels of JA and its derivatives as well as biosynthesis and signaling components change in response to stress are still missing in potato. Therefore, a deeper understanding of regulatory factors is required, particularly the crosstalk with other hormones and the impact on plant growth. However, a tight modulation of JA metabolism seems like a promising target for future engineering of abiotic stress tolerance in potato (Bittner et al. 2022).

### Extreme sensitivity to waterlogging in potato—integrative -omics highlights commonalities with drought

Our study provides detailed insight into the molecular responses underlying the high vulnerability of potato to waterlogging. Water saturation imposes rapid oxygen deficiency in the soil, thus impairing root respiration and function. Plant survival in flooded soils involves various morphological and metabolic responses to either escape or cope with hypoxia, which involve acclimation responses in roots but also in aerial organs (Sauter 2013; Leeggangers et al. 2023).

The data showed that plant growth and performance were more drastically affected by waterlogging as compared with H, D, and HD treatments. In addition, HTP data suggest that waterlogging had a dominant effect even when applied after a previous combined exposure to heat and drought (HDW; Figs. 1C and 2). When applied as a single stress, detrimental effects on plant performance increased over time. Waterlogging dramatically impairs root conductance and water and nutrient uptake, causing tissue dehydration and wilting. This triggers water-saving responses such as stomatal closure and epinasty, which were reflected in increased leaf temperatures and reduced plant compactness, respectively (Figs. 1 and 2E, Supplementary suppl. Fig. 1). Epinastic leaf movement, a common waterlogging response in *Solanaceae*, is thought to reduce photosystem damage by irradiation and transpiration (Jackson and Campbell 1976; Geldhof et al. 2023). Both stomatal conductance and epinasty are regulated by the pivotal flooding signal ethylene (Leeggangers et al. 2023). While ethylene levels were not measured here, the analyses of synthesis genes (i.e.: *ACO2)* suggested the activation of ethylene production in waterlogged shoots. Ethylene is also known to trigger *RBOH* expression and can act synergistically with ABA to reduce stomatal conductance (Zhao et al. 2021). ABA is also considered to signal root stress during waterlogging (Jackson and Hall 1987; Zhao et al. 2021). We observed both the activation of ABA signaling and ABA accumulation, which together with increased levels of proline, indicates a strong drought signature (Figure 6, A, B, and E). While paradoxical, waterlogging is known to elicit shoot drought responses. As root function in hypoxic soil ceases, it triggers a “drought-like” response in the shoot with the similar goal to trigger water saving measures. A focus on this ABA and drought-mediated regulatory network might thus be an attractive target for probing common resilience mechanisms to both drought and waterlogging.

The energy shortage caused by waterlogging also leads to significant changes in sugar metabolism. The accumulation of soluble sugars such as glucose and fructose might be a consequence of sink-source imbalances during waterlogging and thereby, a decline in shoot-to-root sugar transport. Strikingly, after 1 day of waterlogging, we observed upregulation of *SP6A,* a positive regulator of tuberization, thus suggesting potential roles of this gene in short-term responses to waterlogging.

Prolonged exposure to waterlogging revealed several aspects of late responses and factors contributing to potato susceptibility to waterlogging. Leaves of waterlogged plants overcome energy shortages by recycling carbon from amino acids and GABA. The latter plays an important role not only in TCA replenishment but also in ion homeostasis and reduction of oxidative stress (Lothier et al. 2020; Wu et al. 2021). We observed a strong increase in free amino acids that, together with the upregulation of POP2 and an aminopeptidase (Fig. 6), suggests increased protein breakdown and utilization of amino acids as alternative energy sources. Furthermore, the downregulation of RPL27 (and other ribosomal proteins) could indicate the shutting down of energy-demanding processes, such as protein synthesis, as a response to this energy shortage.

Potato susceptibility to prolonged waterlogging was evidenced by other multi-level events such as the upregulation of proteins related to protein and cell wall component turnover, *RBOHA* upregulation and photosynthesis impairment (Fig. 2, Supplementary Fig. S3, Fig. 6). It is also explained by increased ABA signaling and biosynthesis and *RBOHA* expression, which convergently indicate increased tissue dehydration and oxidative stress that is reflected in the HTP data (Figs. 1C and 2). This includes decreased tuber number and weight, indicating a retardation of both tuber initiation and bulking. As tuberization is a particularly energetically expensive process, the imposition of root zone hypoxia likely disrupts the underground sink force essential for stolon development, tuber initiation and bulking.

Altogether, our data suggest that 2 weeks of waterlogging led to near-lethal effects, and even if acclimation responses were activated, overall, they could not compensate for maintaining root function (i.e. unrecovered water consumption, Supplementary Table S4) and general plant survival, even during recovery, thus confirming the high susceptibility of potato to waterlogging.

## Conclusions

The present comprehensive approach produced a rich integrated dataset, which enabled diverse exploration of molecular mechanisms across various levels and processes. Through the connection of phenotype to molecular responses, we attained deeper insights into the intricate regulation of metabolic and phenotypic traits. This should now guide the identification of key regulators that govern the interplay between molecular dynamics and their phenotypic expressions. The utilization of both knowledge-based approaches and multivariate statistical methods played a crucial role in deciphering complex molecular regulatory networks and their association with phenotypic and physiological traits, thereby facilitating the rapid generation of hypotheses.

In addition to several insights into potato stress responses, this study also provides a blueprint for performing and analyzing single and multiple stress and effective integration of large datasets for potato. Importantly, this setup can also be applied to other plant species. These advancements hold significant implications for potato breeding strategies, providing a deeper understanding of plant stress responses and expediting trait selection. As agricultural landscapes confront challenges like climate change and population growth, embracing multi-omics integration holds promise for cultivating resilient potato varieties that can thrive in various conditions.

## Materials and methods

### Plant growth conditions and sampling

One hundred and fifty in vitro potato cuttings (*Solanum tuberosum* cv. Désirée) were cultivated and grown as described in the Supplementary methods. After 32 days of cultivation, plants were randomly distributed into 6 groups (6 plants each) referring to control group and 5 different stress conditions (heat, drought, combined heat and drought, waterlogging and combination of heat, drought and waterlogging) (Fig. 1). Plants were moved into 2 growth units of Growth Capsule (PSI; [Photon Systems Instruments], Czech Republic) where climate conditions for day/night temperature were set in 1 unit to 22/19 °C, referring to control conditions, and in the second unit to 30/28 °C, referring to heat conditions. In both units growing light intensity was set at 330 *µ*mol m^−2^ s^−1^ PPFD and relative humidity was maintained at 55%. All plants were measured under control in day 0 then the stress treatments depicted schematically in Fig. 1. The treatments were applied as the following: (1) Control conditions—cultivation at 22/19 °C, watering up to 60% FC; (2) Drought conditions—cultivation at 22/19 °C, watering up to 60% FC until day 7, then reduce watering to 30% FC for 1 week (until day 14); (3) Heat conditions—cultivation at 30/28 °C for 2 weeks, watering up to 60% FC until day 14; (4) Heat + Drought conditions—cultivation at 30/28 °C for 2 weeks, watering up to 60% FC for 1 week (until day 7), then reduce watering to 30% FC for 1 week (until day 14); (5) Waterlogging conditions—cultivation at 22/19 °C, watering up to 130% FC for 2 weeks (until day 14); (6) Heat + Drought + Waterlogging conditions—cultivation at 30/28 °C for 2 weeks with watering up to 60% FC for 1 week (until day 7), then reduce watering to 30% FC until day 14 followed by inducing waterlogging by cultivation at 22/19 °C for 1 week with watering up to 130% FC until day 21. Except for Heat + Drought + Waterlogging conditions, all stress treatments were followed by 1 week of recovery (from day 15 and until day 21) in control conditions.

Plants were divided into 2 sets, “phenotyping plants” and “plants for tissue harvest” (see Supplementary Table S1). Phenotyping set consisted of 6 replicates per treatment, in total 36 plants, and was used for daily image-based phenotyping (for definition of scored traits, see Supplementary Table S2).

### High-throughput phenotyping

Prior to the stress treatment initiation and during the stress treatments, all plants were daily phenotyped. A comprehensive phenotyping protocol was used for the acquisition of physiological and morphological traits according to the described method (Abdelhakim et al. 2024). All imaging sensors for digital analysis are being implemented in the PlantScreen™ Modular system (PSI, Czech Republic). The photosynthesis-related traits were determined using kinetic chlorophyll fluorescence imaging where the selected protocol for measuring plants was similar to the defined approach (Abdelhakim et al. 2024). The measurement of temperature profiles of the plants was measured using thermal imaging, where the acquisition and segmentation of the images were processed as described in (Abdelhakim et al. 2021a, 2021b; Findurová et al. 2023). The morphological and growth dynamics were determined using both top and multiple angles (0°, 120°, and 240°) side view RGB imaging, and images were processed as described by Awlia et al. (2016). Each pot was loaded onto a transport disk automatically moving on a conveyor belt between the automatic laser height measuring unit, acclimation unit, robotic-assisted imaging units, weighing and watering unit, and the cultivation greenhouse located area. The raw images were automatically processed and parameters were extracted through PlantScreen^TM^ Analyzer software (PSI, Czech Republic) (Supplementary Table S2). Statistical evaluation was performed to check the differences between the treatments using Wilcoxon test (Supplementary Table S3).

### Tissue sampling

Leaf sampling was conducted on days 1, 7, 8, 14, 15, and 21 after stress treatment initiation (Treatment days). The 2nd and 3rd fully developed leaves were harvested and flash-frozen in liquid nitrogen. Subsequently, leaf tissue was homogenized, aliquoted, and distributed for individual follow-up proteomics, and targeted transcriptomics, metabolomics, and hormonomics analyses. Remaining above ground tissue was harvested and total fresh weight (FW) and dry weight (DW) was measured (Supplementary Table S4). In total, 112 plants with 4 replicates per sampling time and per treatment were collected. The 4th leaf was harvested to calculate relative water content (RWC) (Supplementary Table S4), and 3 leaf disks were collected and weighed, then soaked in water to determine the turgor weight (TW) and dried in the oven to calculate RWC (Supplementary Table S2). In addition, at the end of the experiment, the below ground tissue was collected, where number of tubers per plant and total weight were assessed from 4 replicates per treatment. Harvest index was calculated as a ratio between tuber weight and the total biomass.

### Multi-omics analysis

#### Transcriptomic marker analysis

RT-qPCR was performed to assess the expression of 14 marker genes involved in redox homeostasis, hormonal signaling (ethylene, cytokinin, ABA, SA, and JA), heat stress, tuber development, circadian clock, and calcium signaling using previously validated reference genes (Supplementary Table S7).

RNA was extracted and DNase treated using Direct-zol RNA Miniprep Kit (Zymo Research, USA) from 80 to 100 mg of frozen homogenized leaf tissue, followed by reverse transcription using High-Capacity cDNA Reverse Transcription Kit (Thermo Fisher, USA). The expression of the target and reference genes was analyzed by RT-qPCR, as described previously (Petek et al. 2014). QuantGenius (http://quantgenius.nib.si) was used for quality control, standard curve-based relative gene expression quantification, and imputation of values below level of detection or quantification (LOD, LOQ; Baebler et al. 2017).

#### Hormonomics

Concentration of the endogenous abscisate metabolites, auxin metabolites, jasmonates, and salicylic acid were determined in 10 mg of frozen homogenized leaf tissue according to the method described (Floková et al. 2014) and modified by Široká et al. (2022; see Supplementary methods for details). All experiments were repeated as 4 biological replicates.

#### Metabolomics

For determination of soluble sugar, starch, and amino acid contents, 30–50 mg of freeze-dried leaf or tuber material were extracted with 1 mL of 80% (v/v) ethanol. Soluble sugar and starch content was determined as described in Hastilestari et al. (2018), while amino acids sample preparation and measurements were performed as described elsewhere (Obata et al. 2020; Smith and Zeeman 2006).

#### Proteomics

High-throughput shotgun proteomics was done according to Hoehenwarter et al. (2008) with following modifications: 40 mg of leaf tissue from multiple stress conditions were freeze-dried in liquid N_2_ and ground using mortar and pestle. The proteins were extracted, pre-fractionated (40 *µ*g of total protein were loaded onto the gel [1D SDS-PAGE], trypsin digested, and desalted (using a C18 spec plate)] according to a previously described method (Chaturvedi et al. 2013; Ghatak et al. 2016). One microgram of purified peptides was loaded onto a C18 reverse-phase analytical column (Thermo Scientific, EASY-Spray 50 cm, 2 *µ*m particle size). Separation was achieved using a 2-and-a-half-hour gradient method, starting with a 4% to 35% buffer B (v/v) gradient (79.9% ACN, 0.1% formic acid [FA], 20% ultra-high purity water [MilliQ] over 90 min). Buffer A (v/v) consisted of 0.1% FA in high-purity water (MilliQ). The flow rate was set to 300 nL/min. Mass spectra were acquired in positive ion mode using a top-20 data-dependent acquisition (DDA) method. A full MS scan was performed at 70,000 resolution (m/z 200) with a scan range of 380 to 1800 m/z, followed by an MS/MS scan at 17,500 resolution (m/z 200). For MS/MS fragmentation, higher energy collisional dissociation (HCD) was used with a normalized collision energy (NCE) of 27%. Dynamic exclusion was set to 20 s.

Raw data were searched with the SEQUEST algorithm present in Proteome Discoverer version 1.3 (Thermo Scientific, Germany) described previously (Chaturvedi et al. 2015; Ghatak et al. 2020). Pan-transcriptome (Petek et al. 2020) protein FASTA was employed. The identified proteins were quantitated based on total ion count and normalized using the normalized spectral abundance factor (NSAF) strategy (Paoletti et al. 2006).

### Data analysis

The programming environments R v.4.3 and v4.4 (https://www.r-project.org/) and Python v3.8 (www.python.org) were used. Experimentally acquired data are available from the Supplementary Table S4. All data, code, and algorithms, required for supporting, generating, or reproducing the findings of this study are openly available in GitHub repository at https://github.com/NIB-SI/multiOmics-integration.

### Data preprocessing

A master sample description metadata file was constructed (Supplementary Table S1). Potential inconsistencies between replicates were examined using pairwise plots between omics levels, multidimensional scaling plots, and scatterplot matrices within omics' levels using the vegan v2.6.-4 R package (Oksanen et al. 2022). Missing values were handled as described in the Supplementary methods. Due to many missing values, the neoPA (hormonomics) variable was excluded from further analysis.

Variable selection was conducted on the non-invasive phenomics variable sets (Supplementary Table S4). The random forest (RF) algorithm from the R package caret v6.0-94 (Kuhn 2008) as well as the python package scikit-learn v1.2.0 were used with default settings, as RF showed the best performance out of a selection of algorithms. Recursive feature elimination was applied in R and multiple importance scores, including mutual information, ANOVA, RF importance and SHAP values (Lundberg and Lee 2017) were computed in Python, showing consistencies between the approaches for the top 5 variables (top area, compactness, qL_Lss, ΔT, water consumption; nonredundancy ranking in R). The sixth variable (F_v_/F_m__Lss) was selected based on expert knowledge.

Gene set enrichment was performed on the proteomics dataset using GSEA v4.3.2 (Subramanian et al. 2005) and in-house generated gene sets (Supplementary File 3, Table S6) and visualized using biokit v0.1.1. Proteomics differential expression was conducted using the DEP v 1.22.0 package (Zhang et al. 2018 ) (Supplementary Table S5). For downstream proteomics analyses, differentially abundant and enriched proteins (from pathways important for this experimental setup) were used. Waterlogging stress was cut-off at 1-week duration, while triple stress (HDW) was not considered in downstream analyses due to poor plant performance.

### Analysis of individual omics data layers

Pearson correlation coefficient (PCC) heatmaps (pheatmap v1.0.12, heatmaply v. 1.5.0) were generated within each treatment and for explicit treatment duration. Permutation-based t-test (MKinfer v1.2) was used to denote differences between specific treatment and control within the corresponding time-point (Kohl, 2024). Corresponding log2FC were calculated. For downstream analyses, 4 out of 6 replicates were chosen from non-invasive phenomics measurements to allow integration with invasive phenomics and other omics measurements conducted on 4 replicates.

### Integration across different omics datasets

Correlations between components measured in various Omics' levels were calculated and visualised using DIABLO (Singh et al. 2019) as implemented in the mixOmics v6.24.0 package (Rohart et al. 2017). The correlation matrix was calculated separately for each stress as well as for control.

### Integration of data with prior knowledge

A background knowledge network was manually constructed considering biochemical pathways between measured variables. Where necessary, pathways were simplified to only include representative variables, to prevent addition of many unmeasured nodes that would impede the visualization. Proteomics differential expression results were merged with *t*-test and log2FC results (Supplementary Table S3). Final networks were visualized using DiNAR (Zagorscak et al. 2018) and Cytoscape (Shannon et al. 2003).

For additional reports and some results not used in this manuscript, see Supplementary supplementary methods and a project's GitHub repository https://github.com/NIB-SI/multiOmics-integration.

### Accession numbers

Links of gene names used in the study to GeneIDs can be found in Supplementary Table S5 for proteomics data and Supplementary Table S7 for qPCR.

## Supplementary Material

kiaf126_Supplementary_Data

## Data Availability

Experimentally acquired data and data required to reproduce the analysis are available from Supplementary Table S4 and NIBs' GitHub repository https://github.com/NIB-SI/multiOmics-integration. The MS/MS spectra of the identified proteins and their meta-information from both databases have been deposited to the ProteomeXchange Consortium via the PRIDE partner repository (https://www.ebi.ac.uk/pride) with the dataset identifier PXD052587.

## Supplementary data

The following materials are available in the online version of this article.

**Supplementary File S1.** Principal coordinates analysis (PCoA) of Bray-Curtis dissimilarity between samples.

**Supplementary File S2.** Differential Network Analysis animation.

**Supplementary File S3.** Pan-transcriptome Gene Matrix Transposed (GMT) file format.

**Supplementary Figure S1.** Plant morphological responses of control and stress-exposed plants.

**Supplementary Figure S2.** Log2FC of a tuber metabolite's relative abundance between stress and control.

**Supplementary Figure S3.** Correlation analysis within and between omics levels.

**Supplementary Table S1.** Phenodata.

**Supplementary Table S2.** Phenomics featuredata.

**Supplementary Table S3.** Statistical results across different omics levels.

**Supplementary Table S4.** Omics measurements.

**Supplementary Table S5.** Differential protein expression.

**Supplementary Table S6.** Gene set enrichment analysis of proteomics data.

**Supplementary Table S7.** Genes used for transcriptome analysis using RT-qPCR.

**Supplementary Table S8.** CRediT (Contributor Roles Taxonomy) authorship statement.

## Dive Curated Terms

The following phenotypic, genotypic, and functional terms are of significance to the work described in this paper:

POP2 Gramene: AT3G22200
POP2 Araport: AT3G22200

RPL27 Gramene: AT5G40950
RPL27 Araport: AT5G40950

HSP70 Gramene: AT3G12580
HSP70 Araport: AT3G12580

ACO2 Gramene: AT1G62380
ACO2 Araport: AT1G62380

DPA Gramene: days post-anthesis
DPA Araport: days post-anthesis

PIF4 Gramene: Solyc07g043580
PIF4 Araport: Solyc07g043580

CAT1 Gramene: Solyc12g094620
CAT1 Araport: Solyc12g094620

ERF1 Gramene: Acc25620
ERF1 Araport: Acc25620

RWC Gramene: Relative Water Content
RWC Araport: Relative Water Content

HSP101 Gramene: AT1G74310
HSP101 Araport: AT1G74310

proteins CHEBI: CHEBI:36080
