# Supplementary material for: Integration of multi-omics data and deep phenotyping provides insights into responses to single and combined abiotic stress in potato
Source: Plant Physiol. 2025 Apr 2;197(4):kiaf126. doi: 10.1093/plphys/kiaf126 (PMC12012603; doi:10.1093/plphys/kiaf126)
Supplement: kiaf126_Supplementary_Data [file kiaf126_supplementary_data.zip › Supplementary Data.pdf]

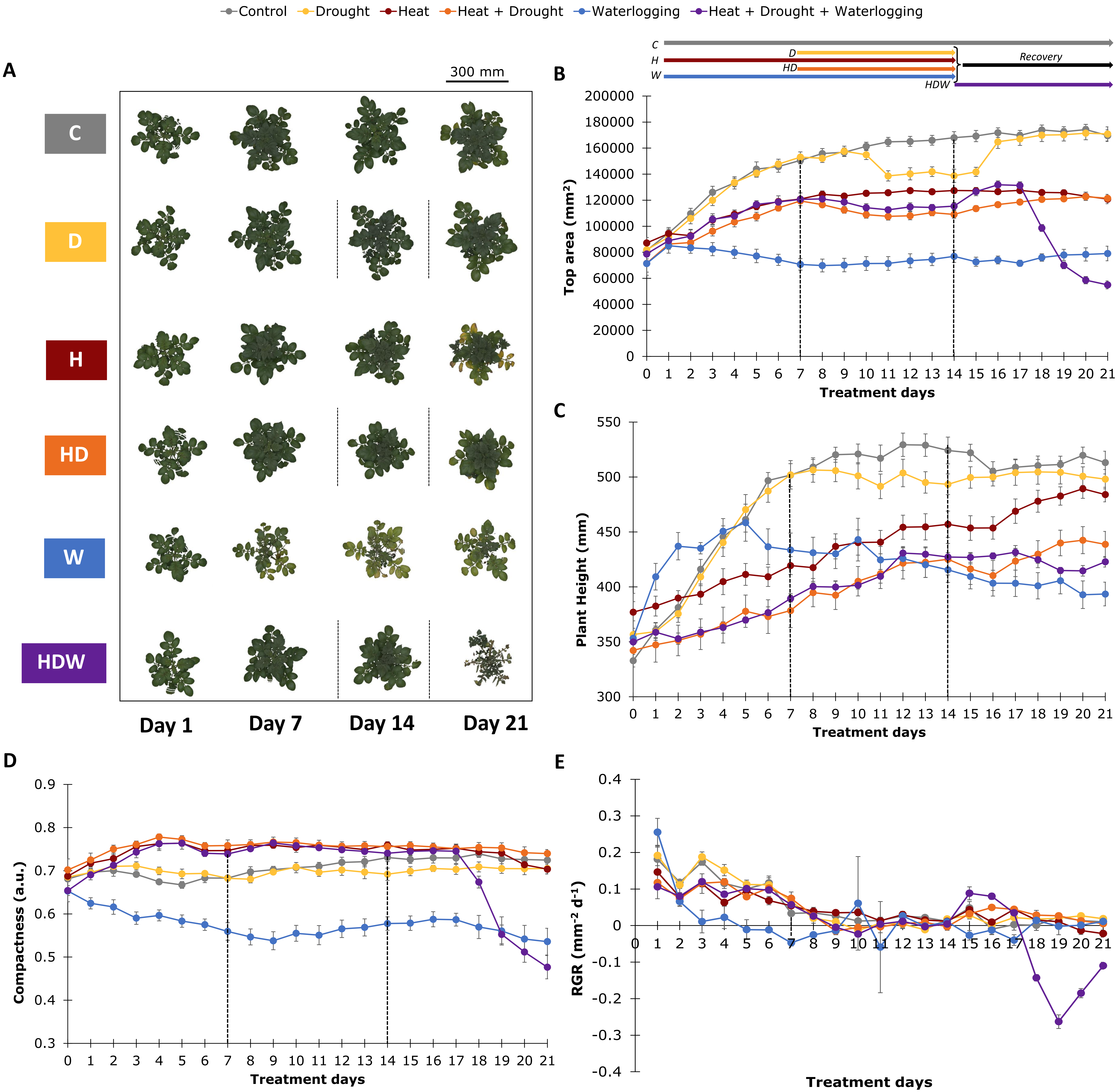

Control
Drought
Heat
Heat + Drought
Waterlogging
Heat + Drought + Waterlogging
A
300 mm
C
D
H
HD
W
HDW
Day 1
Day 7
Day 14
Day 21
B
C
D
H
HD
W
HDW
Recovery
Top area (mm²)
Treatment days
C
Plant Height (mm)
Treatment days
D
Compactness (a.u.)
Treatment days
E
RGR (mm⁻² d⁻¹)
Treatment days

Supplementary Figure S1: Plant morphological responses of control and stress-exposed plants. (A) Top view RGB images at selected time points of tissue sampling starting from day 1 where i) in the first week heat stress was induced for H, HD, HDW marked rows, then ii) in the second week drought was induced for D, HD, HDW up to day 14, and finally iii) in the third week waterlogging was induced for HDW up to day 21, while the other treatments were recovered. Images were digitally extracted for comparison. (B-E) Parameters based on analyzed RGB images; Top area, plant height, compactness, and relative growth rate (RGR). The data represent mean values ± standard error of mean (n = 6). C: control, D: individual drought stress, H: individual heat stress, HD: combined drought with heat stress, W: individual waterlogging stress, HDW: triple-stress condition.

	D28	H28	HD28	W28
Glucose	-0.520	**0.886**	**1.026**	-0.363
Fructose	0.185	-0.362	0.497	0.708
Sucrose	0.014	0.019	0.480	**2.050**
Starch	0.115	**-0.419**	**-0.289**	**-0.563**
Ala	-0.137	**-0.731**	-0.437	0.919
Arg	-0.291	**-0.710**	-0.262	**1.588**
Asn	-0.124	-0.508	-0.187	**2.802**
Asp	-0.107	-0.038	0.164	**1.230**
Gln	-0.056	-0.562	-0.098	**2.611**
Glu	-0.225	**-0.404**	**-0.299**	0.091
Gly	-0.300	**-0.846**	**-0.568**	**1.045**
His	**-0.428**	**-0.664**	-0.322	**1.546**
Ile	-0.250	**-0.542**	-0.323	**1.001**
Leu	-0.298	-0.295	-0.075	**1.017**
Lys	**-0.477**	**-0.876**	**-0.753**	0.434
Met	-0.239	**-0.817**	**-0.485**	**0.408**
Phe	-0.389	**-1.437**	**-1.090**	**-1.158**
Pro	-0.241	-0.131	0.028	2.850
Ser	-0.146	**-0.595**	-0.374	**1.447**
Thr	-0.083	**-0.775**	-0.365	**1.189**
Tyr	-0.361	**-0.824**	**-0.742**	-0.059
Val	-0.141	**-0.640**	-0.301	**0.963**

	D28	H28	HD28	W28
Number oftubers	+26%	**+32%**	+16%	**-82%**
Totaltubersweight	-4.6%	**-48%**	**-48%**	**-97%**

Supplementary Figure S2: Log2FC of a tuber metabolite's relative abundance between stress and control. Numbers in bold correspond to comparisons with p-value < 0.05. Additionally, log2FC for number of tubers and total tuber weight is shown.

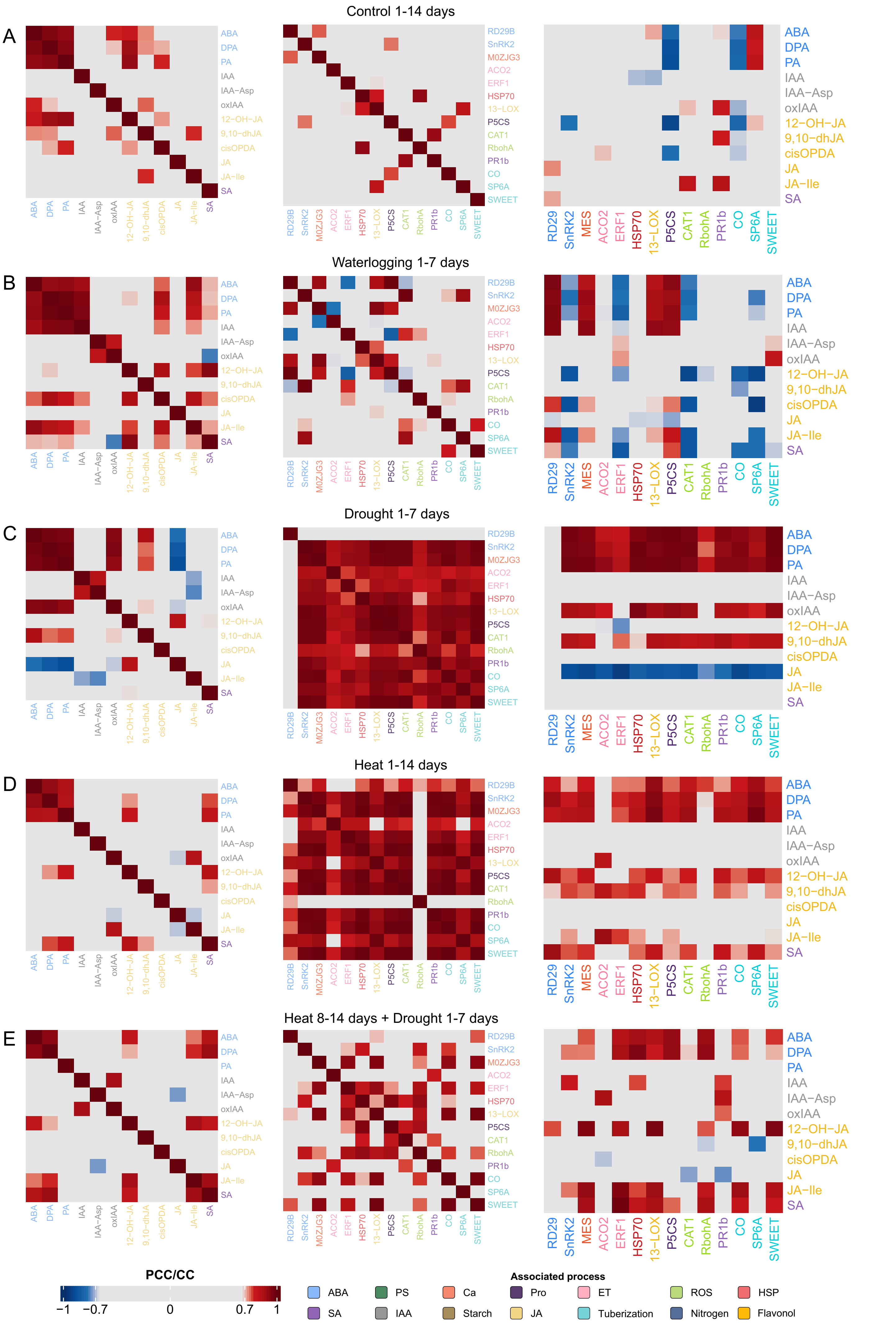
Control 1-14 days
Waterlogging 1-7 days
Drought 1-7 days
Heat 1-14 days
Heat 8-14 days + Drought 1-7 days
A
B
C
D
E
ABA
DPA
PA
IAA
IAA−Asp
oxIAA
12−OH−JA
9,10−dhJA
cisOPDA
JA
JA−Ile
SA
RD29B
SnRK2
M0ZJG3
ACO2
ERF1
HSP70
13−LOX
P5CS
CAT1
RbohA
PR1b
CO
SP6A
SWEET
RD29
MES
PCC/CC
−1
−0.7
0
0.7
1
Associated process
ABA
PS
Ca
Pro
ET
ROS
HSP
SA
IAA
Starch
JA
Tuberization
Nitrogen
Flavonol

Supplementary Figure S3: Correlation analysis within and between omics levels. A) Two weeks of control condition, B) one week of waterlogging, C) one week of drought, D) two weeks of heat, E) one week of heat combined with one week of drought. For correlation analysis within omics levels (left panel: hormones, middle panel: transcripts) heatmaps display Pearson correlation coefficient (PCC). For analysis between components of different molecular levels (right panel: hormones vs transcripts) heatmaps display canonical correlation analysis (CCA) results. Variable prioritisation for CCA was conducted using multiblock sPLS-DA (Singh et al., 2019).
