# Supplementary material for: Integration of multi-omics data and deep phenotyping provides insights into responses to single and combined abiotic stress in potato
Source: Plant Physiol. 2025 Apr 2;197(4):kiaf126. doi: 10.1093/plphys/kiaf126 (PMC12012603; doi:10.1093/plphys/kiaf126)
Supplement: kiaf126_Supplementary_Data [file kiaf126_supplementary_data.zip › Supplementary File S1.html]

rglWebGL


3D plot
