# Supplementary material for: Integration of multi-omics data and deep phenotyping provides insights into responses to single and combined abiotic stress in potato
Source: Plant Physiol. 2025 Apr 2;197(4):kiaf126. doi: 10.1093/plphys/kiaf126 (PMC12012603; doi:10.1093/plphys/kiaf126)
Supplement: kiaf126_Supplementary_Data [file kiaf126_supplementary_data.zip › Supplementary Methods.docx]

# Supplementary methods

Contents

[Supplementary methods 1](#_Toc182412791)

[Plant growth conditions 2](#_Toc182412792)

[Multi-omics analysis 2](#_Toc182412793)

[Transcriptomic marker analysis 2](#_Toc182412794)

[Hormonomics 3](#_Toc182412795)

[Metabolomics 3](#_Toc182412796)

[Proteomics 3](#_Toc182412797)

[Data analysis 5](#_Toc182412798)

[Data preprocessing 5](#_Toc182412799)

[Analysis of individual omics data layers 6](#_Toc182412800)

[Integration across different omics datasets 6](#_Toc182412801)

[Automated graph thresholding 7](#_Toc182412802)

[Integration of data with prior knowledge 7](#_Toc182412803)

[References 8](#_Toc182412804)

## Plant growth conditions

150 in-vitro potato cuttings (Solanum tuberosum cv Desirée) were cultivated in fully saturated 250 ml pots filled with 95 g of commercial peat substrate (Klassman 2, Germany) under controlled conditions. 2-weeks old seedlings were transplanted into 3L PSI (Photon Systems Instruments) pots filled with 1600 g of the same substrate, mixed with sand in a 3:1 proportion. The soil surface was covered with blue rubber mat. The climate conditions in the Walk-in FytoScope growth chamber (PSI, Czech Republic) during cultivation were set at 22/19 °C for day/night temperature with 60% relative humidity (RH) and growing light intensity at 330 µmol m-2 s-1 photosynthetic photon flux density (PPFD) (55% cool-white LED and 85% far-red LED lighting as determined with SpectraPen MINI (PSI, Czech Republic)). The plants were grown under long-day conditions (16 h photoperiod) and were regularly watered to maintain soil relative water content at 60% field capacity (FC).

## Multi-omics analysis

### Transcriptomic marker analysis

RT-qPCR was performed to assess the expression of 14 marker genes involved in redox homeostasis (RBOHA, CAT1), hormonal signalling - ethylene (ACO2, ERF1), abscisic acid (P5CS, SnRK2.9, RD29B), cytokinin and salicylic acid (PR1b), jasmonic acid (13-LOX), heat stress (HSP70), tuber development (SWEET, SP6A), circadian clock (CO) and calcium signalling (MES, methyl esterase, Marc Knight, unpublished data), using EF1 and COX as previously validated reference genes (Supp. Table 7).

RNA was extracted and DNase treated using Direct-zol RNA Miniprep Kit (Zymo Research, USA) from 80-100 mg of frozen homogenised leaf tissue, followed by reverse transcription using High-Capacity cDNA Reverse Transcription Kit (Thermo Fisher, USA). The expression of the target and reference genes was analysed by qPCR, as described previously (Petek et al., 2014). QuantGenius ([http://quantgenius.nib.si](http://quantgenius.nib.si/)), was used for quality control, standard curve-based relative gene expression quantification and imputation of values below level of detection or quantification (LOD, LOQ) (Baebler et al., 2017).

### Hormonomics

The samples of 10 mg were extracted in cold 1 mL of 1 mol/L formic acid in 10% aqueous methanol with the addition of internal stable isotope-labelled standards (5 pmol of [^13^C_6_]-IAA, [^13^C_6_]-oxIAA, [^13^C_6_]-IAA-Asp, [^13^C_6_]-IAA-Glu and [^2^H_2_]-(-)-JA-Ile, 10 pmol of [^2^H_5_]-OPDA, [^2^H_6_]-JA and [^2^H_6_]-ABA, and 20 pmol of [^2^H_4_]-SA) and whole extracts were purified on Oasis HLB solid phase extraction columns (1cc/30 mg, Waters) following the described protocol (Flokova et al., 2014). The analysis was performed on a 1260 Infinity II LC/SFC hybrid system, coupled to an Agilent 6495B Triple Quadrupole LC/MS system (Agilent Technologies, Santa Clara, CA, USA) using the settings described (Siroka et al., 2022).

### Metabolomics

For determination of soluble sugar, starch and amino acid contents, 30 - 50 mg of freezed-dried leaf or tuber material were extracted with 1 ml of 80% (v/v) ethanol and incubated at 80°C for 60 min. After centrifugation for 5 min at 13,000 rpm, cleared supernatants were transferred into new tubes and evaporated to dryness at 40°C. The residue was resolved in 300 µl of water and used for the determination of soluble sugars and amino acids. The pellet was used to determine the starch content. The pellet was subsequently incubated with 0.2 M KOH at 95°C for 1 h. The pH value was adjusted to 5.5 by adding 1 M acetic acid and the starch was digested to glucose by treatment with amylogucosidase (1 mg/ ml in 50 mM sodium acetate buffer) overnight. The amount of soluble sugars was determined photometrically as described previously (Smith and Zeeman, 2006). For the determination of amino acid contents, 10µl of extracts were mixed with 80 μl borate buffer (200 mM, pH 8.8) and derivatized with 10μl aminoquinolyl-N-hydroxysuccinimidyl carbamate (AQC) solution by heating it 55°C for 10 min. Subsequently the samples were measured and quantified using standard samples on a Dionex P680-HPLC system with an RF 2000 fluorescence detector (Dionex, Sunnyvale, CA, USA) as described elsewhere (Obata et al., 2020).

### Proteomics

For high-throughput analysis a shotgun proteomics method (Hoehenwarter et al., 2008) was performed with modifications. 40 mg of leaf tissue from multiple stress conditions were freeze-dried in liquid N_2_ and ground using mortar and pestle. The proteins were extracted, pre-fractionated (40µg of total protein were loaded onto the gel (1D SDS-PAGE), trypsin digested and desalted (using a C18 spec plate) according to a previously described method (Chaturvedi et al., 2013; Ghatak et al., 2016). Prior to mass spectrometric measurement, the tryptic peptide pellets were dissolved in 4% (v/v) acetonitrile, 0.1% (v/v) formic acid. One µg of each sample (3 biological replicates for each cell type) was loaded onto a C18 reverse-phase analytical column (Thermo scientific, EASY-Spray 50 cm, 2 µm particle size). Separation was achieved with a two-and-a-half-hour gradient method and was set to 4 - 35% buffer B (v/v) [79.9% ACN, 0.1% formic acid (FA), 20% Ultra high purity (MilliQ)] for 90 minutes and then to 90% buffer B over one minute, and remained constant for an additional 8 minutes. The buffer A (v/v) was 0.1% FA in high purity water (MilliQ). The flow rate was set to 300 nL min−1. LC eluent was then introduced into the mass spectrometer (Q-Exactive Plus, Thermo Scientific) through an Easy-Spray ion source (Thermo Scientific). The emitter was operated at 1.9 kV. The mass spectra were measured in positive ion mode applying a top 20 data-dependent acquisition (DDA). The full MS was set to 70,000 resolution at m/z 200 [AGC target at 3e6, maximum injection time (IT) of 50 ms and a scan range 380-1800 (m/z)]. The full MS scan was followed by a MS/MS scan at 17,500 resolution at m/z 200 [Automatic Gain Control (AGC) target at 5e4, 1.2 m/z isolation window and maximum IT of 100 ms]. For MS/MS fragmentation, normalized collision energy (NCE) for higher energy collisional dissociation (HCD) was set to 27%. Dynamic exclusion was at 20 s. Unassigned and +1, +7, +8 and > +8 charged precursors were excluded. The intensity threshold was set to 1.0e4. The isotopes were excluded

Raw data were searched with the SEQUEST algorithm present in Proteome Discoverer version 1.3 (Thermo Scientific, Germany) described previously (Chaturvedi et al., 2015; Ghatak et al., 2020) . Pan-transcriptome (Petek et al., 2020) protein FASTA was employed and peptides were matched against database plus decoys, considering a significant hit when the peptide confidence was high, which is equivalent to a false discovery rate (FDR) of 1%, and the Xcorr threshold was established at 1 per charge (2 for +2 ions 3 for +3 ions, etc.). The variable modifications were set to acetylation of the N-terminus and methionine oxidation, with a mass tolerance of 10 ppm for the parent ion and 0.8 Da for the fragment ion. The number of missed and non-specific cleavages permitted was two. There were no fixed modifications, as dynamic modifications were used. The identified proteins were quantitated based on total ion count and normalised using the normalised spectral abundance factor (NSAF) strategy (Paoletti et al., 2006).

## Data analysis

The programming environments R v.4.3 (<https://www.r-project.org/>) and Python v3.8 ([www.python.org](http://www.python.org)) were used. Experimentally acquired data and data required to reproduce the analysis are available from Supplemental Table 4 and NIB’s GitHub repository (<https://github.com/NIB-SI/multiOmics-integration>) were used.

### Data preprocessing

A master sample description metadata file was constructed (Supp. Table 1). Potential inconsistencies between replicates were examined using pairwise plots between omics levels, multidimensional scaling plots and scatterplot matrices within omics’ levels using the vegan v2.6-4 R package (Oksanen et al., 2022). Phenomics variable *deltaTemperature (*ΔT*)* was scaled by its minimal value to obtain nonnegative values for consecutive computing. Missing values in phenomics variable *water consumption* at day 1 were replaced by first day nonnegative values, i.e. measurements from day 3. In waterlogging application, pot weight was not measured within 24 hours in day 2 and 4 as water was added to maintain the level of treatment, thus imputation was performed for missing days when calculating water use efficiency (WUE). Missing values in other omics’ levels were imputed using random forest nonparametric missing value imputation with the missForest v1.5 (Stekhoven and Buhlmann, 2012) package. Due to many missing values, the neoPA (hormonomics) variable was excluded from further analysis.

Variable selection was conducted on the non-invasive phenomics variable sets (Supp. Table 4). The random forest (RF) algorithm from the R package caret v6.0-94 (Kuhn, 2008) as well as the python package scikit-learn v1.2.0 were used with default settings, as RF showed the best performance out of a selection of algorithms (see assay <_A_multiOmics-FS-Py> at <https://github.com/NIB-SI/multiOmics-integration)>. Recursive feature elimination was applied in R and multiple importance scores, including mutual information, Anova, RF importance and SHAP values (<https://arxiv.org/abs/1705.07874>) were computed in Python, showing consistencies between the approaches for the top 5 variables: top area (normalised area of foreground pixels according to real-world coordinates), compactness (calculated as the ratio between area and surface of convex hull enveloping particular plan), qL_Lss (steady-state fraction of open reaction centers in PSII in light ), ΔT (averaged temperature of foreground pixels for steady-state temperature normalised to ambient temperature), and water consumption. The sixth variable, F_v_/F_m__Lss (maximum efficiency of PSII photochemistry in the light), was selected based on expert knowledge.

Gene set enrichment was performed on the proteomics dataset using GSEA v4.3.2 (Subramanian et al., 2005) and an in-house generated gene sets (Supp. File 3, Supp. Table 6). Proteomics differential expression was conducted using the DEP v 1.22.0 package (Zhang et al., 2018) (Supp. Table 5). For downstream proteomics analyses, differentially abundant and enriched (pathways important for this experimental setup) proteins were used. Waterlogging stress was cut-off at one-week duration, while triple stress (HDW) was not considered in downstream analyses due to poor plant performance.

### Analysis of individual omics data layers

Summary statistics (mean, SD, SE; CI) and abundance profiles were visualised using ggplot2 v.3.5.1. Pearson correlation coefficient (PCC) heatmaps were generated within each treatment and for explicit treatment duration (psych v. 2.2.9, Hmisc v. 4.8-0, heatmaply v. 1.5.0, corrplot v. 0.95, pheatmap v. 1.0.12, ComplexHeatmap v2.16.0, (Gu et al., 2016). Permutation-based t-test MKinfer v1.2, (Kohl, 2024) was used to denote differences between specific treatment and control within the corresponding time-point. To avoid ties, jitter was added to transcriptomics variables RD29B and SP6A in Heat condition at days 1, 7 and 8. Corresponding log2FC were calculated. For downstream analyses, 4 out of 6 replicates were chosen from non-invasive phenomics measurements to allow integration with invasive phenomics and other omics measurements conducted on 4 replicates.

### Integration across different omics datasets

Correlations between components measured in various Omics’ levels were calculated and visualised using DIABLO (Singh et al., 2019) as implemented in the mixOmics v6.24.0 package (Rohart et al., 2017). The correlation matrix was calculated separately for each stress as well as for control.

Correlations matrices obtained in different stresses were then compared to elucidate both similarities in differences in molecular state of plants under different stresses. This was done by taking the difference in correlation between treatments to obtain a value between -2 (higher in control compared to treatment) and 2 (higher in treatment compared to control). The differential graphs were then thresholded according to an automated thresholding process.

### Automated graph thresholding

Absolute value thresholding was sequentially applied (from 0 to 2, in increments of 0.01). At each increment, edges below the threshold were removed, and subsequently all isolated vertices also removed. At each threshold increment the network structure was studied. Finally, per graph thresholds were defined as the largest threshold resulting in a local minima of the densities, and applied to obtain thresholded networks. <other threshold options if we use them>. Initial thresholding increments were performed in C++ (<https://doi.org/10.5281/zenodo.7614763>) and threshold selection performed in Python.

### Integration of data with prior knowledge

A background knowledge network was manually constructed considering biochemical pathways between measured variables. Where necessary, pathways were simplified to only include representative variables, to prevent addition of many unmeasured nodes that would impede the visualisation. Proteomics differential expression results were merged with t-test and log2FC results (Supp. Table 3) to inspect which parts of the network are responding in particular perturbation and visualised using DiNAR (Zagorscak et al., 2018) and Cytoscape (Shannon et al., 2003).

The background network was also merged with both the thresholded within omics level correlation networks and the thresholded across omics level correlation networks. As in the DiNAR networks, differential expression results were used to colour nodes, in order to display the network response. Network construction was done in Python using networkX and visualisations in Cytoscape (Cytoscape) using the py4cytoscape library.

For additional reports and some results not used in this manuscript see projects GitHub repository <https://github.com/NIB-SI/multiOmics-integration>.

## References

**Baebler S, Svalina M, Petek M, Stare K, Rotter A, Pompe-Novak M, Gruden K** (2017) quantGenius: implementation of a decision support system for qPCR-based gene quantification. BMC Bioinformatics **18:** 276

**Chaturvedi P, Doerfler H, Jegadeesan S, Ghatak A, Pressman E, Castillejo MA, Wienkoop S, Egelhofer V, Firon N, Weckwerth W** (2015) Heat-Treatment-Responsive Proteins in Different Developmental Stages of Tomato Pollen Detected by Targeted Mass Accuracy Precursor Alignment (tMAPA). J Proteome Res **14:** 4463-4471

**Chaturvedi P, Ischebeck T, Egelhofer V, Lichtscheidl I, Weckwerth W** (2013) Cell-specific analysis of the tomato pollen proteome from pollen mother cell to mature pollen provides evidence for developmental priming. J Proteome Res **12:** 4892-4903

**Flokova K, Tarkowska D, Miersch O, Strnad M, Wasternack C, Novak O** (2014) UHPLC-MS/MS based target profiling of stress-induced phytohormones. Phytochemistry **105:** 147-157

**Ghatak A, Chaturvedi P, Bachmann G, Valledor L, Ramsak Z, Bazargani MM, Bajaj P, Jegadeesan S, Li W, Sun X, Gruden K, Varshney RK, Weckwerth W** (2020) Physiological and Proteomic Signatures Reveal Mechanisms of Superior Drought Resilience in Pearl Millet Compared to Wheat. Front Plant Sci **11:** 600278

**Ghatak A, Chaturvedi P, Nagler M, Roustan V, Lyon D, Bachmann G, Postl W, Schrofl A, Desai N, Varshney RK, Weckwerth W** (2016) Comprehensive tissue-specific proteome analysis of drought stress responses in Pennisetum glaucum (L.) R. Br. (Pearl millet). J Proteomics **143:** 122-135

**Gu Z, Eils R, Schlesner M** (2016) Complex heatmaps reveal patterns and correlations in multidimensional genomic data. Bioinformatics **32:** 2847-2849

**Hoehenwarter W, van Dongen JT, Wienkoop S, Steinfath M, Hummel J, Erban A, Sulpice R, Regierer B, Kopka J, Geigenberger P, Weckwerth W** (2008) A rapid approach for phenotype-screening and database independent detection of cSNP/protein polymorphism using mass accuracy precursor alignment. Proteomics **8:** 4214-4225

**Kohl M** (2024) MKinfer: Inferential Statistics. R package **Version 1.2:** <https://github.com/stamats/MKinfer>

**Kuhn M** (2008) Building Predictive Models in R Using the caret Package. Journal of Statistical Software **28:** 1 - 26

**Obata T, Klemens PAW, Rosado-Souza L, Schlereth A, Gisel A, Stavolone L, Zierer W, Morales N, Mueller LA, Zeeman SC, Ludewig F, Stitt M, Sonnewald U, Neuhaus HE, Fernie AR** (2020) Metabolic profiles of six African cultivars of cassava (Manihot esculenta Crantz) highlight bottlenecks of root yield. Plant J **102:** 1202-1219

**Oksanen J, Simpson G, Blanchet FG, Kindt R, Legendre P, Minchin P, hara R, Solymos P, Stevens H, Szöcs E, Wagner H, Barbour M, Bedward M, Bolker B, Borcard D, Carvalho G, Chirico M, De Cáceres M, Durand S, Weedon J** (2022) vegan community ecology package version 2.6-2 April 2022,

**Paoletti AC, Parmely TJ, Tomomori-Sato C, Sato S, Zhu D, Conaway RC, Conaway JW, Florens L, Washburn MP** (2006) Quantitative proteomic analysis of distinct mammalian Mediator complexes using normalized spectral abundance factors. Proc Natl Acad Sci U S A **103:** 18928-18933

**Petek M, Rotter A, Kogovsek P, Baebler S, Mithofer A, Gruden K** (2014) Potato virus Y infection hinders potato defence response and renders plants more vulnerable to Colorado potato beetle attack. Mol Ecol **23:** 5378-5391

**Petek M, Zagorscak M, Ramsak Z, Sanders S, Tomaz S, Tseng E, Zouine M, Coll A, Gruden K** (2020) Cultivar-specific transcriptome and pan-transcriptome reconstruction of tetraploid potato. Sci Data **7:** 249

**Rohart F, Gautier B, Singh A, Le Cao KA** (2017) mixOmics: An R package for 'omics feature selection and multiple data integration. PLoS Comput Biol **13:** e1005752

**Shannon P, Markiel A, Ozier O, Baliga NS, Wang JT, Ramage D, Amin N, Schwikowski B, Ideker T** (2003) Cytoscape: a software environment for integrated models of biomolecular interaction networks. Genome Res **13:** 2498-2504

**Singh A, Shannon CP, Gautier B, Rohart F, Vacher M, Tebbutt SJ, Lê Cao K-A** (2019) DIABLO: an integrative approach for identifying key molecular drivers from multi-omics assays. Bioinformatics **35:** 3055-3062

**Siroka J, Brunoni F, Pencik A, Mik V, Zukauskaite A, Strnad M, Novak O, Flokova K** (2022) High-throughput interspecies profiling of acidic plant hormones using miniaturised sample processing. Plant Methods **18:** 122

**Smith AM, Zeeman SC** (2006) Quantification of starch in plant tissues. Nat Protoc **1:** 1342-1345

**Stekhoven DJ, Buhlmann P** (2012) MissForest--non-parametric missing value imputation for mixed-type data. Bioinformatics **28:** 112-118

**Subramanian A, Tamayo P, Mootha VK, Mukherjee S, Ebert BL, Gillette MA, Paulovich A, Pomeroy SL, Golub TR, Lander ES, Mesirov JP** (2005) Gene set enrichment analysis: a knowledge-based approach for interpreting genome-wide expression profiles. Proc Natl Acad Sci U S A **102:** 15545-15550

**Zagorscak M, Blejec A, Ramsak Z, Petek M, Stare T, Gruden K** (2018) DiNAR: revealing hidden patterns of plant signalling dynamics using Differential Network Analysis in R. Plant Methods **14:** 78

**Zhang X, Smits AH, van Tilburg GB, Ovaa H, Huber W, Vermeulen M** (2018) Proteome-wide identification of ubiquitin interactions using UbIA-MS. Nat Protoc **13:** 530-550
